# Plasma Treatment of PDMS for Microcontact Printing (μCP) of Lectins Decreases Silicone Transfer and Increases the Adhesion of Bladder Cancer Cells

**DOI:** 10.1021/acsami.3c09195

**Published:** 2023-10-27

**Authors:** Joanna Zemła, Renata Szydlak, Katarzyna Gajos, Łukasz Kozłowski, Tomasz Zieliński, Marcin Luty, Ingrid H. Øvreeide, Victorien E. Prot, Bjørn T. Stokke, Małgorzata Lekka

**Affiliations:** † Institute of Nuclear Physics, Polish Academy of Sciences, PL-31342 Krakow, Poland; ‡ M. Smoluchowski Institute of Physics, Jagiellonian University, 30348 Kraków, Poland; § Biophysics and Medical Technology, Department of Physics, The Norwegian University of Science and Technology (NTNU), NO-7491 Trondheim, Norway; ∥ Biomechanics, Department of Structural Engineering, The Norwegian University of Science and Technology (NTNU), NO-7491 Trondheim, Norway

**Keywords:** microcontact printing, PDMS, cell adhesion, ToF-SIMS imaging, lectin micropatterns

## Abstract

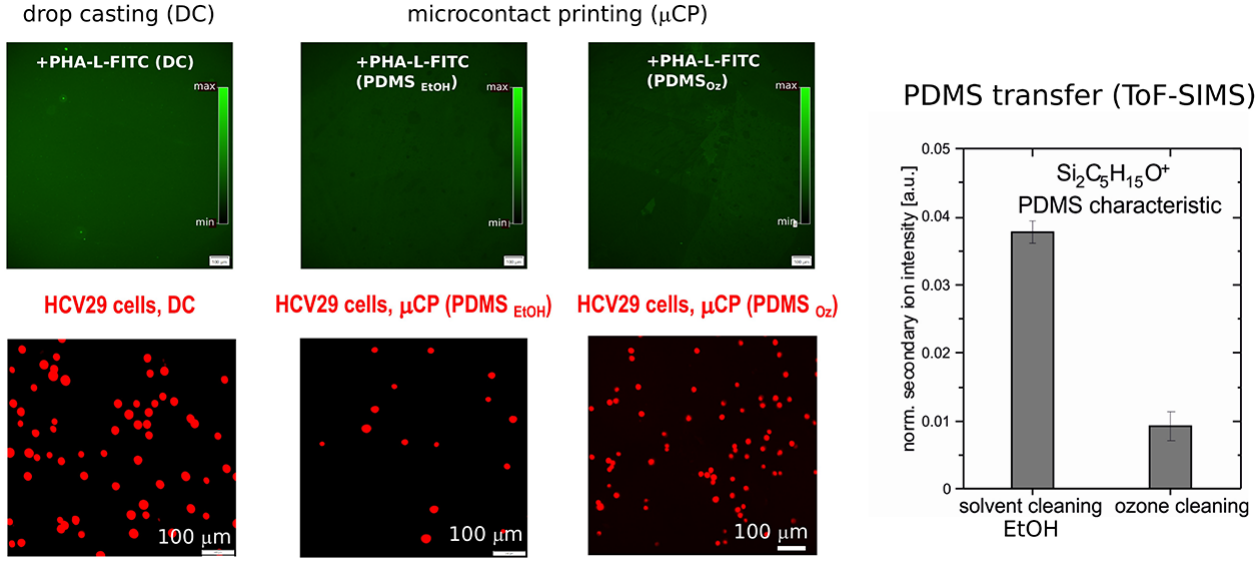

The present study investigates silicone transfer occurring during microcontact printing (μCP) of lectins with polydimethylsiloxane (PDMS) stamps and its impact on the adhesion of cells. Static adhesion assays and single-cell force spectroscopy (SCFS) are used to compare adhesion of nonmalignant (HCV29) and cancer (HT1376) bladder cells, respectively, to high-affinity lectin layers (PHA-L and WGA, respectively) prepared by physical adsorption and μCP. The chemical composition of the μCP lectin patterns was monitored by time-of-flight secondary ion mass spectrometry (ToF-SIMS). We show that the amount of transferred silicone in the μCP process depends on the preprocessing of the PDMS stamps. It is revealed that silicone contamination within the patterned lectin layers inhibits the adhesion of bladder cells, and the work of adhesion is lower for μCP lectins than for drop-cast lectins. The binding capacity of microcontact printed lectins was larger when the PDMS stamps were treated with UV ozone plasma as compared to sonication in ethanol and deionized water. ToF-SIMS data show that ozone-based treatment of PDMS stamps used for μCP of lectin reduces the silicone contamination in the imprinting protocol regardless of stamp geometry (flat vs microstructured). The role of other possible contributors, such as the lectin conformation and organization of lectin layers, is also discussed.

## 1 Introduction

Microcontact printing techniques (μCPs) using PDMS stamps have been applied in many research fields, such as biopatterning,^1−14^ (opto)electronics,^10,15−18^ sensors,^19−22^ materials science,^15,23^ microfluidics,^24^ and others. Low cost and feasibility of the technique have been the reasons for the vast applications of different variations of μCP for decades. Additionally, because of the biocompatibility of PDMS,^25,26^ PDMS-based (bio)patterning has been the flagship approach for substrate modification to aid cell adhesion or impose the direction of growth of cells in a 2D culture.^27−29^

Despite the widespread use of protein patterning with elastomer stamps, little attention has been directed toward optimizing PDMS stamp cleaning protocols. Some publications do not detail PDMS stamp preparation and processing before applying the protein solution, although it has been shown that even when printing with an elastomer stamp just wetted with water, PDMS (silicone) transfer occurs.^30^ The most common approaches of elastomer stamps pretreatment are to sonicate them in pure alcohol, or its water dilution, and later water^1,6,28^ or to use plasma (oxygen, UV ozone, etc.) to activate the PDMS surface,^30−32^ and some combine the two procedures.^11^ Despite there being a growing consensus that PDMS transfer occurs in all cases, there is limited knowledge of the possible influence of silicone contamination on the functionality of biopatterns. Only Foley et al.^1^ investigated the silicone transfer issues concerning protein positioning. They compared the number of bound immunoglobulins (IgGs) to layers of antigens positioned by physical adsorption and μCP finding a significantly lower binding capacity of imprinted biomolecules. X-ray photoelectron spectroscopy (XPS) measurements revealed the presence of silicone oligomers on top of the imprinted antigen layer, which caused a reduction of the protein binding capacity of the biochip. They observed such a silicone transfer and concomitant impact on protein binding despite the fact that the PDMS stamps were sonicated in a 50% solution of isopropanol for 15 min, rinsed with water, and dried with nitrogen (N_2_). Studies on protein binding capacity when UV ozone cleaned/activated PDMS stamps are used for printing are less widespread. Glasmästar et al.^30^ reported that using UV ozone for PDMS stamps reduces silicone contamination of the imprinted surface. They used an elastomer stamp wetted with Milli-Q water on SiOx, TiO_2_, and Au substrates. Interestingly, the XPS and ToF-SIMS analysis showed that silicone-related material is transferred from flat stamps and even more so from patterned stamps.

In this study, we investigated the impact of silicone transfer on the biofunctionality of lectin layers and patterns because of our interest in the interactions of cells with lectin-coated surfaces, as our ultimate goal is to use such surfaces for the detection of bladder cancer cells.^33^ To obtain a detailed analysis of the surface chemistry of biolayers, we use ToF-SIMS. The functionality of the imprinted biomolecules was verified by the adhesion of bladder cancer cells to lectin-coated surfaces. Two protocols of PDMS stamp treatment were tested, i.e., solvent cleaning (sonication of elastomer stamps in ethanol and, subsequently, deionized water) and ozone processing (exposing PDMS stamps to UV ozone plasma). In parallel, we addressed the issue of silicone contamination occurring during the preparation of single- and dual-lectin patterns. We report that pretreatment of PDMS stamps used for μCP of biomolecules can significantly affect the biofunctionality of the obtained patterns. The results indicate that the UV ozone treatment is a remedy for silicone contamination.

## 2 Materials and Methods

### 2.1 Cell Cultures

Bladder cells were cultured in tissue culture flasks 25 cm^2^ (TPP, Genos, Poland) containing an appropriate culture medium in a CO_2_ incubator (Nuaire), providing an optimal culture condition, i.e., 37 °C, 5% CO_2_, and 95% humidity. Non-malignant cell cancer of the ureter (HCV29 cell line) and bladder carcinoma HT1376 cells were grown in Roswell Park Memorial Institute medium 1640 (RPMI 1640, Sigma-Aldrich, Poznań, Poland) and Eagle’s Minimum Essential Medium (EMEM, ATCC LGC Standards, Teddington, Middlesex, U.K.), respectively. All culture media were supplemented with 10% fetal bovine serum (FBS, ATCC LGC Standards, Teddington, Middlesex, U.K.). No antibiotics were used for the cell cultures.

### 2.2 Lectins

Lectins *Phaseolus vulgaris* (PHA-L), wheat germ agglutinin (WGA), fluorescein-labeled PHA-L (excitation/emission 495/515 nm), and rhodamine-labeled WGA (excitation/emission 550/575 nm) were purchased from Vector Laboratories (BIOKOM, Janki, Poland). WGA is a homodimer containing 186 amino acid residues (*M*_n_ = 37.51 kDa, PDB entry 1WGT). PHA-L is a homotetramer (252 amino acid residues, *M*_n_ = 111.02 kDa, PDB entry 1FAT). Both chosen lectins, WGA and PHA-L, belong to the group of plant legume lectins that specifically recognize carbohydrates containing *N*-acetylglucosamine (GlcNAc)^34^ and mannose (Man),^35^ respectively. Each building unit hosts a binding site for one sugar unit. Lectins and the corresponding fluorescently labeled substitutes were dissolved in deionized water (dH_2_O) at a 50 μg/mL concentration.

### 2.3 PDMS Stamps Preparation

The PDMS stamps were realized using photolithography and soft-lithography. First, an Si-wafer master mold was fabricated using photolithography with a negative resist (Mr-DWL5, Microresist Technology, Berlin, Germany). The resist was spin-coated on the wafer to achieve the desired heights and soft-baked following a 50–90 °C temperature ramping protocol. The stamp design was exposed onto the wafer using a maskless aligner (MLA150 Heidelberg-Instruments, Heidelberg, Germany) at 405 nm wavelength and post-exposure baked following the temperature ramping protocol. The wafer rested for 2 h before developing (using MrDev600, Microresist Technology, Berlin, Germany). The wafer was then silanized by following a vacuum-based protocol. Before PDMS (Sylgard 184, Dow, Midland, USA) was poured over the mold at a 1:10 (curing agent:elastomer) ratio, it was gently mixed in plastic cup with a disposable plastic teaspoon for about 3–5 min and desiccated under vacuum for about 15–25 min. Next, the mixture was poured over the master mold and baked at 65 °C for 3 h. Microstructured and flat PDMS stamps were cut and released from the master mold.

### 2.4 Rheological Properties of PDMS

A rheometer (MRC302, Anton Paar, Graz, Austria) equipped with sand-blasted parallel plates (8 mm diameter) was applied for the amplitude sweep measurements. The measurements were performed on PDMS disks of 1.1 mm in thickness and 8.0 mm in diameter at a frequency of 1 Hz and shear strains 0.01–100%. Storage (*G′*) and loss (*G″*) moduli were determined. A wider deformation range allows the upper limit of the linear viscoelastic range to be found (deformation range for which *G*′ and *G*″ are independent of deformation).

### 2.5 Determination of the Sol Fraction and Swelling Ratio

The sol fraction of the cured PDMS was determined using solvent extraction of free molecules released to the immersing cyclohexane phase. Thus, three PDMS samples were placed in 25 mL of cyclohexane (Sigma-Aldrich, Poznań, Poland) for 24 h. Afterward, they were placed in the oven (VO200, Memmert, Poland) and dried at 65 °C for 3 h. The PDMS samples were weighed at each stage of the process. The sol fraction (*Q*_sol_) was calculated using eq 1:
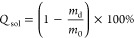
1where *m*_0_ is the initial mass of the PDMS sample while *m*_d_ is its weight after drying. Also, the swelling of PDMS stamps exposed to ethanol was determined. Three elastomer cuboids were fully immersed in ethanol (∼50 mL, Sigma-Aldrich, Poznań, Poland) for 48 h. After 24 h, half of the solvent volume was exchanged. During this time, PDMS samples swelled, and the increased mass and volume were noted. Mass swelling parameter *Q*_m_ was derived from the mass ratio before (*m*_0_) and after swelling (*m*_s_). Finally, all elastomer cuboids were dried in the oven (*T* = 65 °C, *t* = 3 h), and *Q*_sol_ was calculated.

### 2.6 PDMS Treatment for μCP

Two protocols for PDMS stamp cleaning were applied in our study. First, referred herein to the solvent cleaning, PDMS stamps were sonicated in ethanol (EtOH) for 15 min, then in dH_2_O for 15 min and finally dried with a N_2_ stream. We refer to the EtOH-cleaned PDMS as PDMS_EtOH_ in the following. The other method, ozone cleaning, uses a UV ozone cleaner (L2002A2, Ossila, The Netherlands) working with ambient air. Here, the stamp was cleaned with a dust-free tissue soaked with 70% EtOH and then with dH_2_O. After additional drying in a stream of N_2_, PDMS stamps were placed in the UV ozone cleaner for 3 min. The stamps cleaned this way are referred to as PDMS_Oz_ in the following. Both PDMSEtOH and PDMS_Oz_ were immediately used to deposit lectins on the chosen substrates (glass or cell culture Petri dish).

### 2.7 Preparation of Substrates (Glass Slides and Petri Dishes)

Microscope glass slides (Heinz Herenz Hamburg, Germany) were cut into 1 × 1 cm^2^ pieces, cleaned by sonication in EtOH (15 min), followed by sonication in dH_2_O (15 min). The glass slides were subsequently dried with a stream of N_2_ and treated in the UV ozone cleaner for 3 min. Next, they were modified with (3-aminopropyl)triethoxysilane (APTES, Sigma-Aldrich, Poznań, Poland) by the vapor-phase deposition method.^36^ For single-cell force spectroscopy (SCFS), Petri dishes (TPP, Genos, Poland) were functionalized with APTES similarly to the glass slides.

### 2.8 Preparation of Lectin-Coated Surfaces

Lectins were deposited by a drop-casting (DC) method or μCP. In the DC approach, an 80 μL drop of lectin solution was deposited on a glass slide for 30 min. Next, the sample was rinsed with phosphate buffered saline (PBS, Sigma-Aldrich, Poznań, Poland) to remove unbound molecules. For μCP, flat PDMS stamps were used. The surface of freshly cleaned PDMS stamps was covered with a 100 μL drop of the lectin solution. After 15 min, the stamp was rinsed 3 times with PBS and gently dried with a N_2_ stream. Then, the PDMS stamp was placed on the APTES-modified glass surface and pressed gently for 10–20 s. The same procedure was applied in the case of SCFS measurements (PDMS_EtOH_). Here, cast and imprinted domains of lectins were located in different but marked areas of the Petri dish (TPP, Genos, Poland). Both lectin-modified glass slides or Petri dishes were placed in PBS and kept at 4 °C until further use.

### 2.9 Fabrication of Lectin Micropatterns

PDMS stamps (patterned with 40 μm wide parallel lines 80 μm apart and of 50 μm × 100 μm) were used for single and dual lectin pattern fabrication. Single-lectin micropatterns are those composed of only one type of lectin. They were prepared by μCP of a lectin directly on the APTES-functionalized substrate, and dual-lectin patterns were prepared by μCP of a lectin on the protein layer previously deposited on the APTES-functionalized substrate by the DC method. The selectivity and quality of the lectin patterns were verified by a static adhesion assay and ToF-SIMS in imaging mode.

### 2.10 Static Adhesion Assay

Bladder cells were grown in culture flasks (Sarstedt, Nümbrecht, Germany) at 37 °C, 5% CO_2_, and 95% humidity to reach 90% confluency. Before the static adhesion assay, cells were detached using a solution of 0.25% trypsin–EDTA (Sigma-Aldrich, Poznań, Poland) and centrifuged for 4 min at 1800 rpm. Next, cells were resuspended in the corresponding culture medium (4 mL) and counted. 10 mL of cell suspension containing 10^6^ cells/mL was transferred to another tube (15 mL centrifuge tube, TPP, Genos, Poland), and fluorescent dye, CellTracker Red CMTPX (Invitrogen, Thermo Fisher Scientific, Waltham, USA, excitation/emission 577/602 nm) or CellTracker Blue CMAC (Invitrogen, Thermo Fisher Scientific, Waltham, USA, excitation/emission 353/466 nm), was added at 25 μM or 10 μM concentration, respectively. Non-malignant HCV29 cells were labeled with CellTracker Red CMTPX, while cancerous HT1376 cells were labeled with CellTracker Blue CMAC. Tubes with cell suspensions were placed in a CO_2_ incubator for 30 min, then centrifuged, washed in PBS, centrifuged again, and resuspended in 10 mL of the appropriate culture medium. A 1 mL cell suspension (10^5^ cells/mL of HCV29 or HT1376 cells) was subsequently deposited on lectin-modified surfaces (glass) and incubated at 37 °C for 15 min. Then, the substrates were washed three times in PBS to remove unbound cells. A fluorescence microscope was applied to visualize the cells attached to surfaces in three randomly selected locations of each sample. The experiment was repeated three times.

### 2.11 Fluorescence Microscopy and Image Analysis

A fluorescence microscope (Olympus IX83) was used to visualize the lectin patterns and detect the number of adherent cells to the lectin-coated surfaces. Images (obtained using an objective UPLFLN2 10×/0.3, Olympus) were recorded either by an ORCA-spark (Hamamatsu Instruments, Hamamatsu, Japan) camera (image resolution 1920 px × 1200 px) or by a Photometrics Prime BSI Express camera (image resolution 2048 px × 2048 px). The field of view was 1.2 mm × 0.77 mm and 1.3mm × 1.3 mm, respectively. A 100 W mercury lamp was used for fluorescence excitation, and a set of filters was applied to record the fluorescent images of the appropriate dyes. A U-FBW filter was applied to record the fluorescein emission. TRITC and CellTracker Red emissions were collected with a U-FGW filter, while CellTracker Blue emissions were visualized with a U-FUW filter. All images were acquired using CellSens software (Olympus) and processed with ImageJ (version 1.53k, https://imagej.nih.gov/ij/). Cells were counted with a MatLab (version R2022a) script using basic morphological functions.

### 2.12 Time-of-Flight Secondary Ion Mass Spectrometry (ToF-SIMS)

Chemical imaging and molecular composition analysis of lectin patterns and accompanying reference samples were performed using a ToF-SIMS 5 (ION-TOF GmbH) instrument. The Bi^3+^ clusters, produced by a 30 keV Bi liquid metal ion gun, were used as the primary ions. The ion dose density was lower than 10^12^ ions/cm^2^ to ensure static mode conditions. A low-energy electron flood gun was used for charge compensation. High-resolution mass spectra of positive ions were recorded from several areas on lectin patterns and also on uniform reference samples (APTES modified glass, one-component WGA, and PHA-L DC layers) with area sizes of 500 μm × 500 μm (512 × 512) and 200 μm × 200 μm (256 × 256 points), respectively. Mass calibration was performed with H^+^, H_2_^+^, CH^+^, C_2_H_2_^+^ and C_4_H_5_^+^ peaks, while the mass resolution (*m*/Δ*m*) was >6000 at C_4_H_5_^+^. In the case of patterned samples, spectra were reconstructed selectively from the areas of printed patterns and the background during the data analysis. The size of areas from which spectra were reconstructed was kept to 40 000 μm^2^ equal to planar reference samples (200 μm × 200 μm). The intensities of selected peaks from each spectrum were normalized to the total ion intensity for the PDMS transfer examination and to the sum of amino acids derived ions for the lectin composition examination. Additionally, ToF-SIMS images of positive and negative ions were acquired in image mode from several 500 μm × 500 μm areas of lectin pattern samples (the data collection was conducted on a 512 × 512 grid).

### 2.13 Single-Cell Force Spectroscopy (SCFS)

Commercially available tipless cantilevers (Arrow-TL1, NanoWorld) were exposed to a UV ozone plasma cleaner for 3 min. Next, they were placed in APTES vapors for 2 h and immersed in the 2% glutaraldehyde (Sigma-Aldrich, Poznań, Poland) solution in dH_2_O for 30 min at room temperature (RT). The cantilevers were subsequently immersed in aqueous concanavalin A (Con A; 2 mg/mL in PBS, incubated 1 h at RT), washed with the buffer (3 times), and stored in PBS at 4 °C. Before measurements, all functionalized cantilevers were calibrated. The average spring constant was 0.044 ± 0.012 N/m (*n* = 9 cantilevers), and the average photodetector sensitivity was 71.7 ± 12.8 nm/V (*n* = 9).

Con A-functionalized probes were used to immobilize a single bladder cell (either HCV29 or HT1376 cells) at the end of the cantilever in the following way. First, a functionalized cantilever was mounted on the tip-holder of the AFM and immersed in a Petri dish filled with the appropriate culture medium (without FBS). The bottom surface of the Petri dish was modified with lectin(s) by DC or μCP as described above. Then, a 50 μL drop of cell suspension was added to the medium (close to the edge of the Petri dish). Next, the cantilever was placed over the selected cell, and the force–distance curve was collected at a velocity of 5 μm/s and 5 s contact time. Then, the Con A-functionalized cantilever with a cell attached was withdrawn for 15 min above the surface. An inverted optical microscope (Olympus IX72), combined with AFM, was used to control the position of the sample and cantilever and the shape of the probing cell.

SCFS measurements were realized with a CellHesion head (atomic force microscope (AFM), Bruker-JPK, Berlin, Germany). Detachment (adhesion) events between single bladder cancer cells (HCV29 or HT1376) and lectin domains (PHA-L or WGA) produced by DC and μCP were measured. Force–distance curves were collected at a velocity (approach and retract) of 8 μm/s. The loading force was 3 nN. The cell–surface contact time was 0.25 and 3.0 s for HCV29 and HT1376 cells, respectively. This was optimized for each cell line to obtain a sufficiently long baseline of force–distance curves (we do not compare HCV29 to HT1376 data). In each case, a grid of 8 × 8 over 100 μm^2^ was acquired. 10 cell probes were used per case resulting in 640 force–distance curves used for analysis. The adhesion strength was quantified as the adhesion work (*W*_adh_) needed to detach a single cell from the lectin-coated surface (Figure S1) as described elsewhere.^33^ Data were analyzed using JPK Data Processing Software (Bruker-JPK, Berlin, Germany).

### 2.14 Statistical Analysis

Mass and volume swelling ratios and sol fraction were expressed as the mean ± SD. Distributions of the number of adhered cells and adhesion work are presented as box plots and expressed as the mean ± SD or median (interquartile range (IQR), which is defined as the difference of the third (Q_3_) and first (Q_1_) quartiles), respectively. Data were tested with one-way ANOVA with Tukey post-hoc test at the *p* = 0.05 significance threshold. Statistical differences are quantified with *p*-values: *p* < 0.05 (*), *p* < 0.01 (**), *p* < 0.001 (***), and *p* < 0.0001 (****). The analysis was performed with OriginPro2017 software.

## 3 Results

### 3.1 Rheological Properties of PDMS Stamps

**Figure 1:**
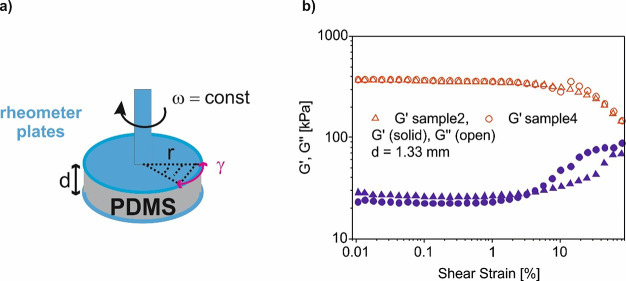
Mechanical properties of PDMS stamps used in this study. (a) Schematic illustration of amplitude sweep measurement (*d*, measuring gap; *r*, plate diameter; ω, oscillation frequency; γ, shear deformation). (b) Exemplary results for the two measured PDMS stamps. Amplitude sweeps were performed at a frequency of 1 Hz, and deformations were in the range of 0.01–100%. Storage (*G*′) and loss (*G*″) moduli depend on the shear deformation.

For μCP of various molecules, especially lectins, the chemical and physical properties of PDMS stamps are crucial for the quality of the imprinted patterns. One of the parameters causing undesirable application failure is the rigidity of the elastomer stamp. When PDMS is too soft, stamp deformations such as buckling and roof collapse are likely to happen.^37^ For too rigid PDMS stamps, diffusion of the protein solution along the surface often occurs.^37,38^ Commercially available elastomers differ in their mechanical properties. It was also shown that these could change over time.^25,39^ Here, we examined the rheological properties of PDMS cuboids by performing amplitude sweep measurements with an oscillatory rheometer (Figure 1).

For PDMS samples, the obtained shear storage modulus (*G′*) values varied from 375 to 475 kPa, with an overall value of 416 ± 56 kPa. The shear loss modulus (*G″*) was in the range of 10–30 kPa with an overall value of 29.5 ± 7.5 kPa (Figure 1). PDMS is assumed to be an incompressible material. For such materials, Poisson’s ratio is 0.5. Thus, the relation between Young’s *E* (elastic modulus being a material response to compression) and *G′* follows the relation *E* = 3*G*′.^40^ Based on this equation and rheological measurements, we estimated Young’s modulus for PDMS stamps to be about 1.1–1.4 MPa, which is in excellent agreement with *E* = 1.35 MPa reported for PDMS at 10:1 elastomer:cross-linker ratio.^41^ The mechanical properties of PDMS are related to the cross-linking density (ρ_*k*_) by *E* = 1.5 *kT*ρ_*k*_, where *k* is the Boltzmann constant and *T* is the temperature.^39,42,43^ Thus, the stoichiometric ratio of the monomer to hardener and curing conditions (*T* and time (*t*)) determine the final cross-linking density. Moučka et al.^42^ showed that the cross-linking density of PDMS (10:1) was 2.1 × 10^26^ m^–3^ at a curing temperature of 25 °C. With the temperature increase to 100 °C, ρ_*k*_ increased to 5.0 × 10^26^ m^–3^. Further increase in the curing temperature to 150 °C resulted in a cross-linking density of 7.2 × 10^26^ m^–3^. These results indicate a substantial sol fraction when using a curing temperature of 65 °C. Indeed, the sol fraction experiment proves the presence of free silicone chains. Long-time immersion of PDMS stamps in cyclohexane resulted in substantial mass swelling, *Q*_m_ = 1.754 ± 0.023, followed by a 4.5% mass reduction of PDMS stamps after drying compared to the initial mass. Most probably, silicone oligomers diffused from elastomer cuboids to the immersing liquid. The obtained results are in agreement with previously reported data showing that even for high stoichiometric ratios, 5 or higher, the excess of nonbonded PDMS residues from the starting material is about 5%.^44^ In addition, elastomer stamps used in our study exhibited volume and mass swelling, 1.148 ± 0.019 and 1.026 ± 0.003, respectively, when immersed in EtOH. We also found *Q*_sol_ of 3.1% for immersion of the solvent cleaned PDMS_EtOH_ in ethanol. It appears to be high because we know that the Hildebrandt solubility (a numerical estimate of the degree of interaction between materials that is a good indicator of solubility, especially for nonpolar materials such as many polymers) parameters of PDMS and EtOH are incompatible (δ_PDMS_ = 7.3 cal^0.5^ cm^1.5^ and δ_EtOH_ = 12.7 cal^0.5^ cm^1.5^,^20,45^ respectively), and one would not expect EtOH exposure to result in such a mass reduction. This indicates that there is still loosely bound silicone on the PDMS_EtOH_ that can result in PDMS transfer during microcontact printing. This phenomenon has been previously observed,^1,21,23,30,46−50^ and some suggested that postsonication in pure ethanol can be used to remove the transferred oligomers.^50^ On the other hand, others claim that transferred oligomers are not harmful contaminants.^23,47,49^ Only Foley et al.^1^ reported that the contamination impacts the further application of imprinted substrates.

### 3.2 Adhesion of Bladder Cancer Cells to Lectin-Modified Substrates

**Figure 2:**
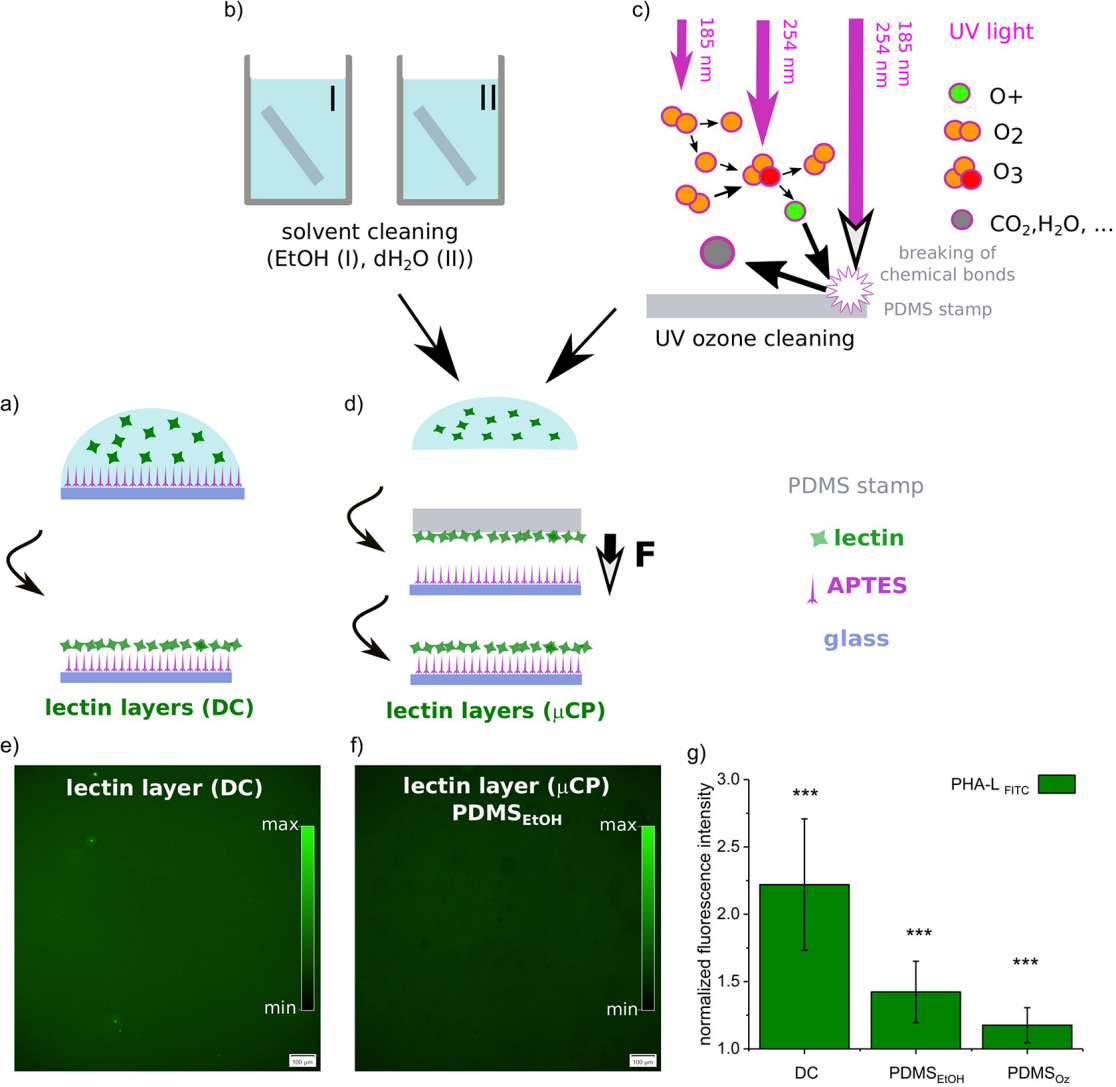
Lectin-modified substrates prepared by drop casting (a) and microcontact printing (b-d). *F* is the normal force applied to a PDMS stamp. Solvent or UV ozone cleaning (c) of PDMS stamps was applied. The mechanism of UV ozone cleaning is schematically illustrated^51^ (c). Fluorescence images (excitation time of 6 ms) of lectin layers prepared by DC (e) and μCP (solvent cleaning, PDMS_EtOH_) (f). (g) Fluorescence intensities of PHA-L layers normalized to the background signal. Data are presented as the mean ± SD. Statistical significance between fluorescence intensity of lectin layers and reference (glass + APTES) was obtained by *t*-test.

To assess the effect of silicone transfer on cell adhesion, the static adhesion of HCV29 and HT1376 to lectin layers prepared by drop casting (Figure 2a,e) and μCP (Figure 2b–d,f) was investigated. The μCP procedure was conducted using both PDMS_EtOH_ and PDMS_Oz_ (Figure 2b,c). Thus, the contaminants on the PDMS surface are diluted with EtOH or removed by the reaction of atomic oxygens and residues of dissociated hydrocarbon contaminants, converting them into simpler volatile molecules H_2_O, CO_2_, and others, which desorb from the surface (Figure 2c) as reported by Kohli et al.^51^ Fluorescence images of the lectin layers prepared by DC and μCP (PDMS_EtOH_) are shown in Figures 2e,f, S2, and S3. Figure 2g presents mean values of the fluorescence intensity normalized to the background signal (APTES coated glass). The highest fluorescence signal was obtained in the case of drop-cast lectin layers (2.22 ± 0.49 and 2.87 ± 0.29 for PHA-L-FITC and WGA-TRITC, respectively). μCP lectin layers emitted fluorescence of lower intensity. We found no significant difference in fluorescence intensity of WGA lectins imprinted with PDMS treated with EtOH, cyclohexane (CHX) (see Supporting Information Figures S2 and S3), or UV ozone. Normalized fluorescence intensity of PHA-L layers imprinted with a UV ozone treated elastomer stamp was 1.18 ± 0.13. For both lectins there was no statistically significant difference in the normalized fluorescence intensity when biomolecules were transferred with PDMS_EtOH_ and PDMS_CHX_ stamps (Figures S2 and S3). In the case of WGA normalized fluorescence intensity values are 2.41 ± 0.65 and 2.03 ± 0.10, respectively, while these were observed for PHA-L to 1.42 ± 0.23 and 1.37 ± 0.26. The lower fluorescence intensity of the lectins suggests a lower number of biomolecules on the surface.^52^ However, fluorescence images confirm an even distribution of lectins over the area examined.

**Figure 3:**
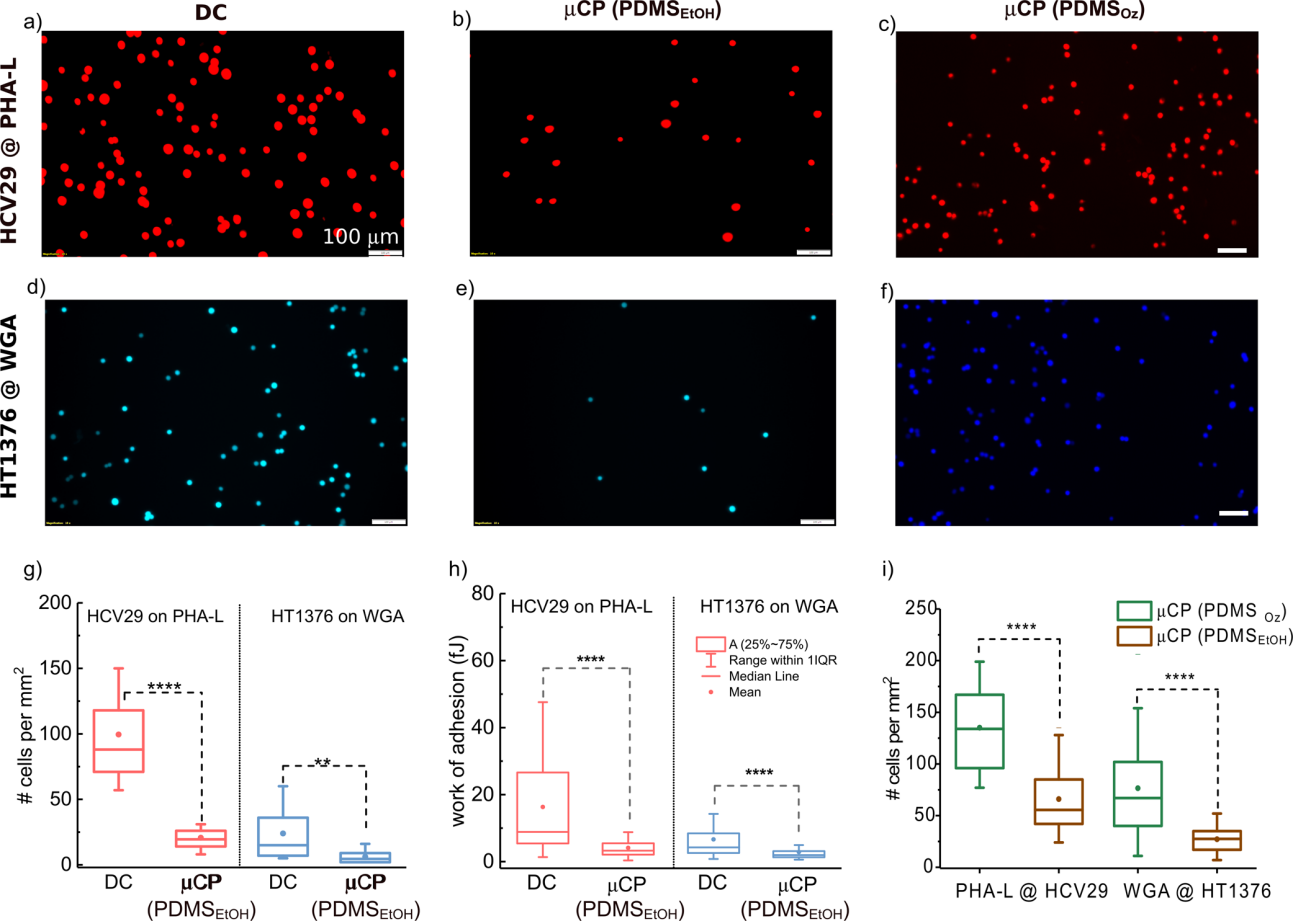
Adhesion of HCV29 and HT1376 cells to PHA-L and WGA-modified substrates prepared using solvent casting or μCP. Exemplary fluorescent images of HCV29 (a–c) and HT1376 (d–f) cells, which adhered to lectins deposited by drop casting (a, d) and μCP with solvent-cleaned (b, e) and ozone-cleaned (c, f) PDMS flat stamps. Cell surface density of bladder cells adhered to substrates prepared by DC and μCP using PDMS_EtOH_ (g). Distributions of the work of adhesion of bladder cancer cells from chosen lectins deposited by DC and μCP using PDMS_EtOH_. The contact time of HCV29 and HT1376 cells with lectins was 0.25 and 3.0 s, respectively (h). (i) Cell surface density of the bladder cells adhered to lectins deposited by μCP (flat PDMS_EtOH_ or PDMS_Oz_). Data are presented as box plots (25–75%). Median (line), mean values (solid dots), and outliers (interquartile range, Q_3_–Q_1_) are shown (g–i). The scale bars are 100 μm (a–f).

To investigate possible effects of PDMS transfer on cell adhesion, we selected lectins based on our previous research, where we studied the interaction of bladder cancer cells with lectin-coated surfaces under static and flow conditions.^33^ Under flow conditions, we found a strong affinity of HCV29 cells to PHA-L, while the adhesion of HCV29 cells to WGA was low (5.15 ± 0.70 cells/mm^2^ vs 1.66 ± 0.38 cells/mm^2^). For HT1376 cells, the highest surface density was observed on the WGA-coated surface, 5.31 ± 0.34 cells/mm^2^, and the lowest on the PHA-L-coated surfaces, at a level of 1–2 cells/mm^2^. These trends were consistent with those observed in the static adhesion assay, so the pairs HCV29 and PHA-L and HT1376 and WGA were selected. Figure 3 presents the results of the adhesion of bladder cancer cells to lectin-modified substrates. Fluorescence microscopy data revealed a significantly lower number of HCV29 cells adhered to PHA-L deposited with PDMS_EtOH_ (Figures 3b and S4) than for DC lectin-coated surface (Figures 3a and S4). HCV29 cells on the drop-casted lectin layer counted 100 ± 37 cells/mm^2^, while on the imprinted (PDMS_EtOH_) layer of PHA-L, a mean of 21 ± 11 cells/mm^2^ was noted (Figure 3g). The same behavior was observed for HT1376 cells. On the imprinted layer of WGA proteins using PDMS_EtOH_ a cell surface density of 6.2 ± 4.5 cells/mm^2^ was observed (Figure 3e,g). Simultaneously, more HT1376 cells adhered (24 ± 21 cells/mm^2^) on the WGA drop-cast layer (see Figure 3d,g). Further, we evaluated the strength of the adhesion of bladder cancer cells to the lectin layers. In Figure 3h, the results of SCFS measurements are presented. It is revealed that in the case of both bladder cancer cell lines, less energy (lower *W*_adh_) is needed to detach a cell from the imprinted lectin layer. In the case of HCV29 cells, *W*_adh_ drops from 8.8 (21) fJ to 3.3 (3.4) fJ. *W*_adh_ obtained when the HT1376 cell was detached from the WGA layer deposited by drop-casting was 4.2 (5.9) fJ. A 46% lower work of adhesion value was noted in the case of the interaction of HT1376 and microcontact printed WGA molecules. The results indicate a significant reduction in cell density and cell adhesion strength toward lectin-coated surfaces prepared by PDMS_EtOH_ relative to drop-cast ones, thus implying a significant reduction of biomolecule functionality associated with the deposition protocol.

**Figure 4:**
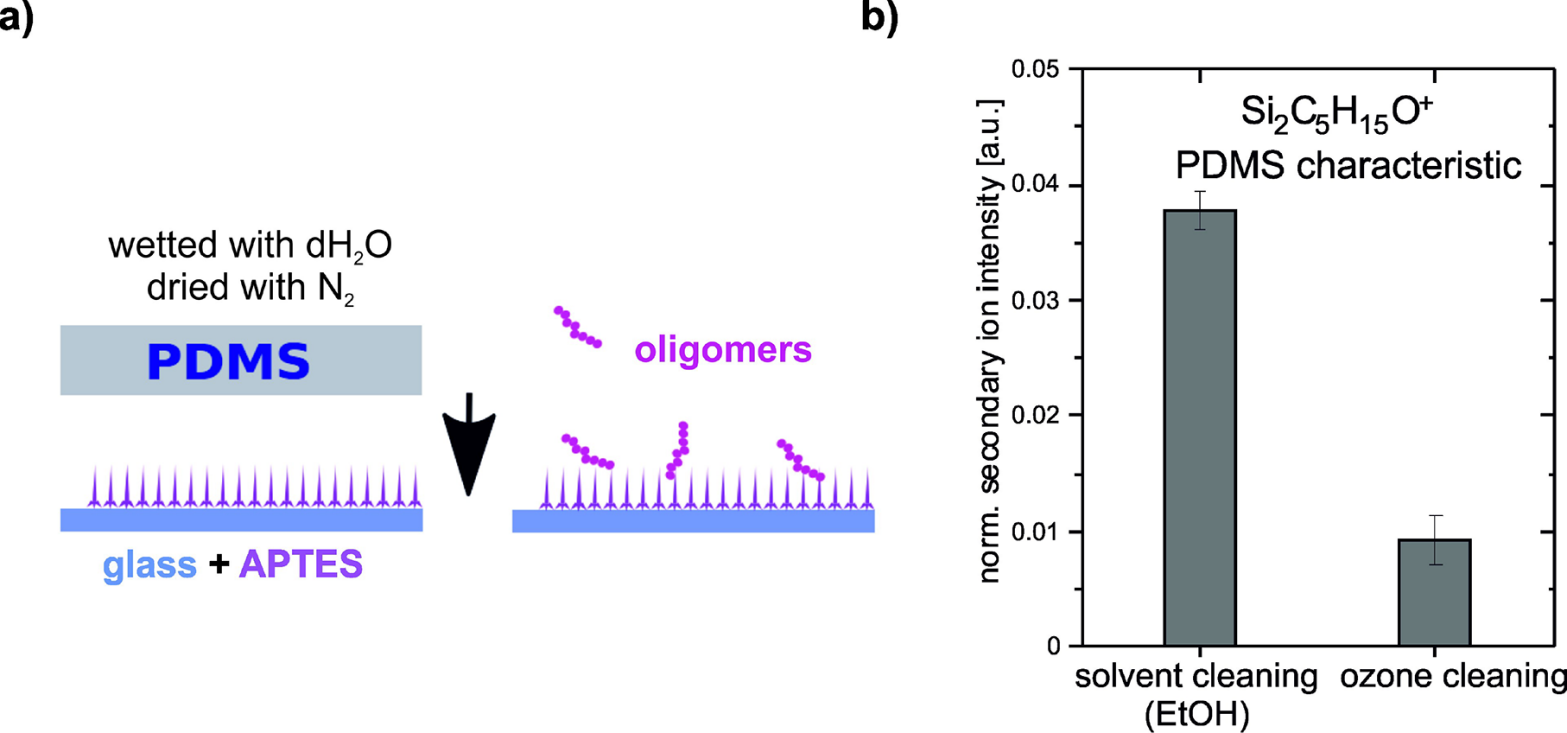
ToF-SIMS characterization of microcontact printed surface obtained by PDMS imprinting APTES coated glass. (a) Schematic illustration of the sample preparation: PDMS stamp (solvent or ozone cleaned) is wetted with dH_2_O, dried with a nitrogen stream, and brought into contact with the APTES coated glass slide. (b) Comparison of PDMS transfer to APTES modified glass surface during imprinting of PDMS stamp, incubated in dH_2_O, after solvent and plasma stamp cleaning. Intensities of Si_2_C_5_H_15_O^+^ (*m*/*z* = 147) ions, characteristic of PDMS, were determined from high mass resolution ToF-SIMS spectra.

The data presented in Figure 3c,f,i show that using PDMS_Oz_ stamps for the lectin deposition improves cell adhesion compared to that obtained by PDMS_EtOH_. The fluorescence micrographs (Figure 3c,f) reveal numerous adhered HCV29 and HT1376 cells, with an overall cell surface density of 135 ± 39 HCV29 cells/mm^2^ adhering to PHA-L lectin deposited by PDMS_Oz_ and 66 ± 36 cells/mm^2^ for lectins deposited by PDMS_EtOH_. The corresponding observation of HT1376 cells adhering to the WGA-TRITC modified substrates was 77 ± 49 and 27 ± 13 cells/mm^2^ for the PDMS_Oz_ and PDMS_EtOH_ stamps, respectively. The above results of cell adhesion experiments indicated that cleaning the PDMS stamps was crucial for the binding capacity of imprinted lectins. The chemical composition of APTES-coated glass slides after contact with solvent- and plasma-cleaned PDMS stamps (Figure 4a) was therefore investigated by ToF-SIMS to provide detailed information on the applied μCP protocol.

Figure 4b shows the intensities of Si_2_C_5_H_15_O^+^ (*m*/*z* = 147) ions characteristic for PDMS,^47^ determined from high mass resolution ToF-SIMS spectra. Microcontact printing using the PDMS_Oz_ as compared to the PDMS_EtOH_ stamps resulted in a significant decrease (∼4-fold) in the intensity of the Si_2_C_5_H_15_O^+^ signals (Figure 4b). This result indicates that PDMS contamination of lectin-modified substrates prepared using the μCP process reduces the adhesion of HCV29 and HT1376 cells.

### 3.3 Quality of Lectin-Based Micropatterns Assessed by Fluorescent Microscopy

**Figure 5:**
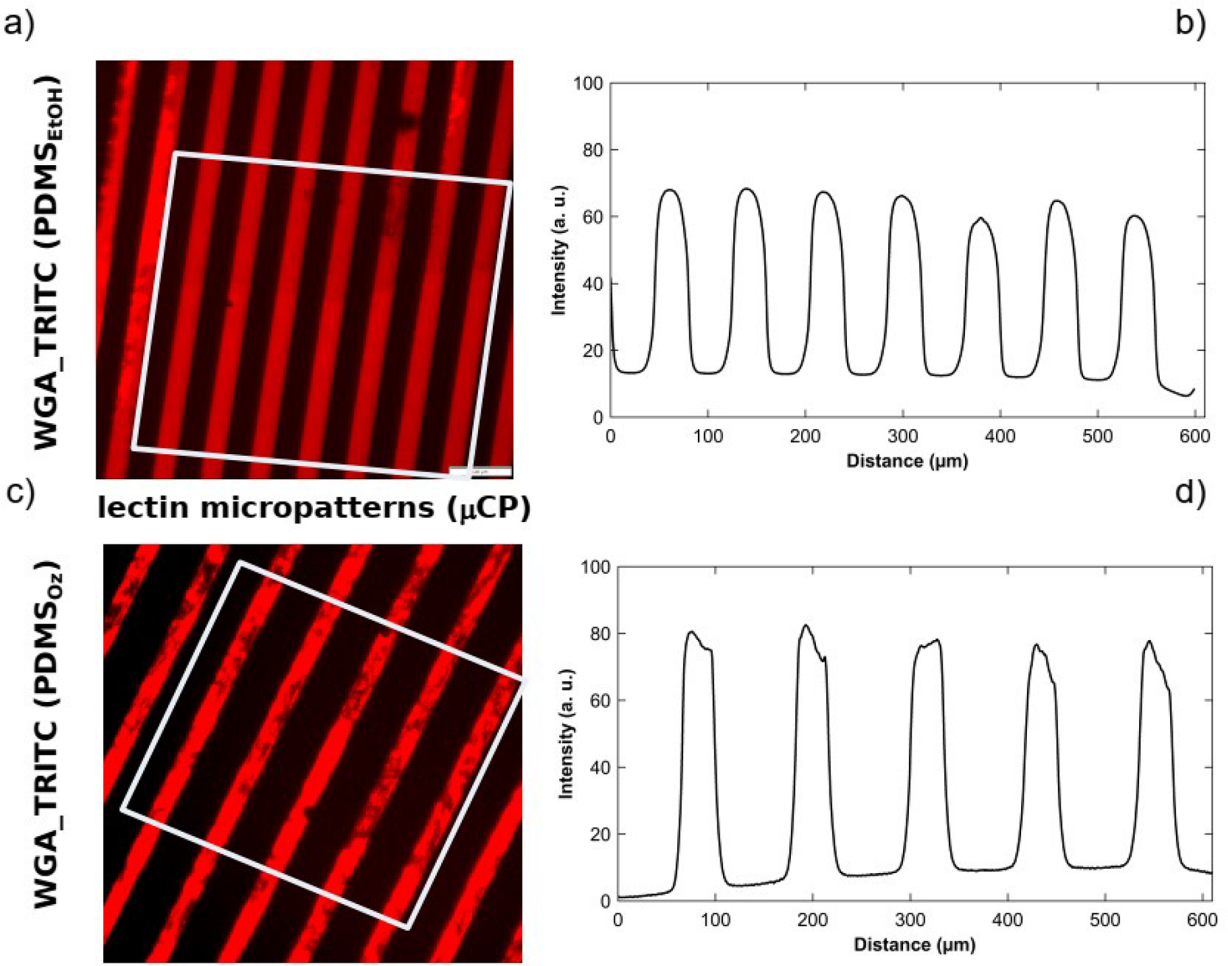
Single-lectin patterns. Representative fluorescence images of WGA-TRITC (red) patterns obtained from solvent (EtOH) (a) and ozone (c) cleaned PDMS stamps, 40 μm × 40 μm and 40 μm × 80 μm, respectively. (b, d) Average of the intensity profiles (perpendicular to striped pattern) obtained from the patterns in (a) and (c) within the areas depicted with white rectangles. For the schematic of the printing process see Figure S7.

While there is limited application potential for homogeneously deposited lectins obtained with a flat PDMS stamp, lectin patterns are very important in biochips and cell patterning. We therefore prepared patterned WGA on glass surfaces using microstructured PDMS stamps (both were cleaned using either solvent (EtOH) or ozone cleaning). A schematic of the printing process is shown in Figure S7. The result is striped areas of WGA-TRITC and APTES. Representative fluorescence images of single-lectin patterns are shown in Figure 5a,c, and quantitative information on the distributions is presented as the average of fluorescence intensity profiles as a function of distance perpendicular to the stripes (Figure 5b,d). A high-intensity signal is obtained from areas in contact with a PDMS stamp for both stamp cleaning protocols.

**Figure 6:**
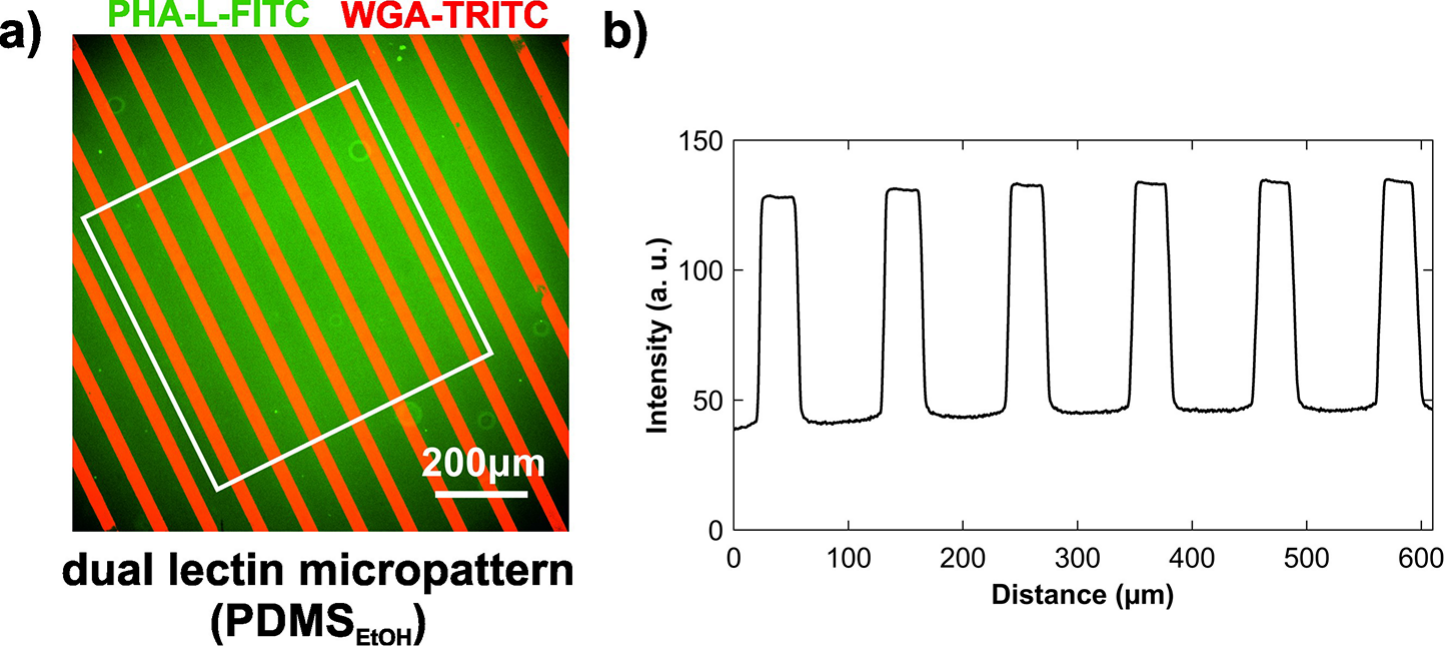
Dual-lectin patterns obtained by μCP (PDMS_EtOH_) of TRITC labeled WGA on initially drop-cast FITC labeled PHA-L on APTES coated glass. (a) Representative overlaid fluorescence micrographs collected for WGA-TRITC (red) and PHA-L-FITC (green). The scale bar is 200 μm. (b) Average of the intensity profiles (perpendicular to striped pattern) obtained from the patterns in (a) within the area depicted with a white rectangle. Schematic of the printing process is shown in Figure S8.

Dual-lectin patterns are required for selective cell capture. We combined DC and μCP approaches (Figure S8) to produce selective dual-lectin patterns. The overlaid fluorescence micrographs of the WGA-TRITC (red) and PHA-L-FITC (green) lectin patterns (Figure 6a) indicate clear stripes of WGA appearing in this final structure. The average of the fluorescence intensity profiles shown in Figure 6b confirms the uniform distribution of lectins in areas with and without contact with a PDMS stamp.

### 3.4 ToF-SIMS Analysis of PDMS Transfer in Single-Lectin and Dual-Lectin Micropatterns

**Figure 7:**
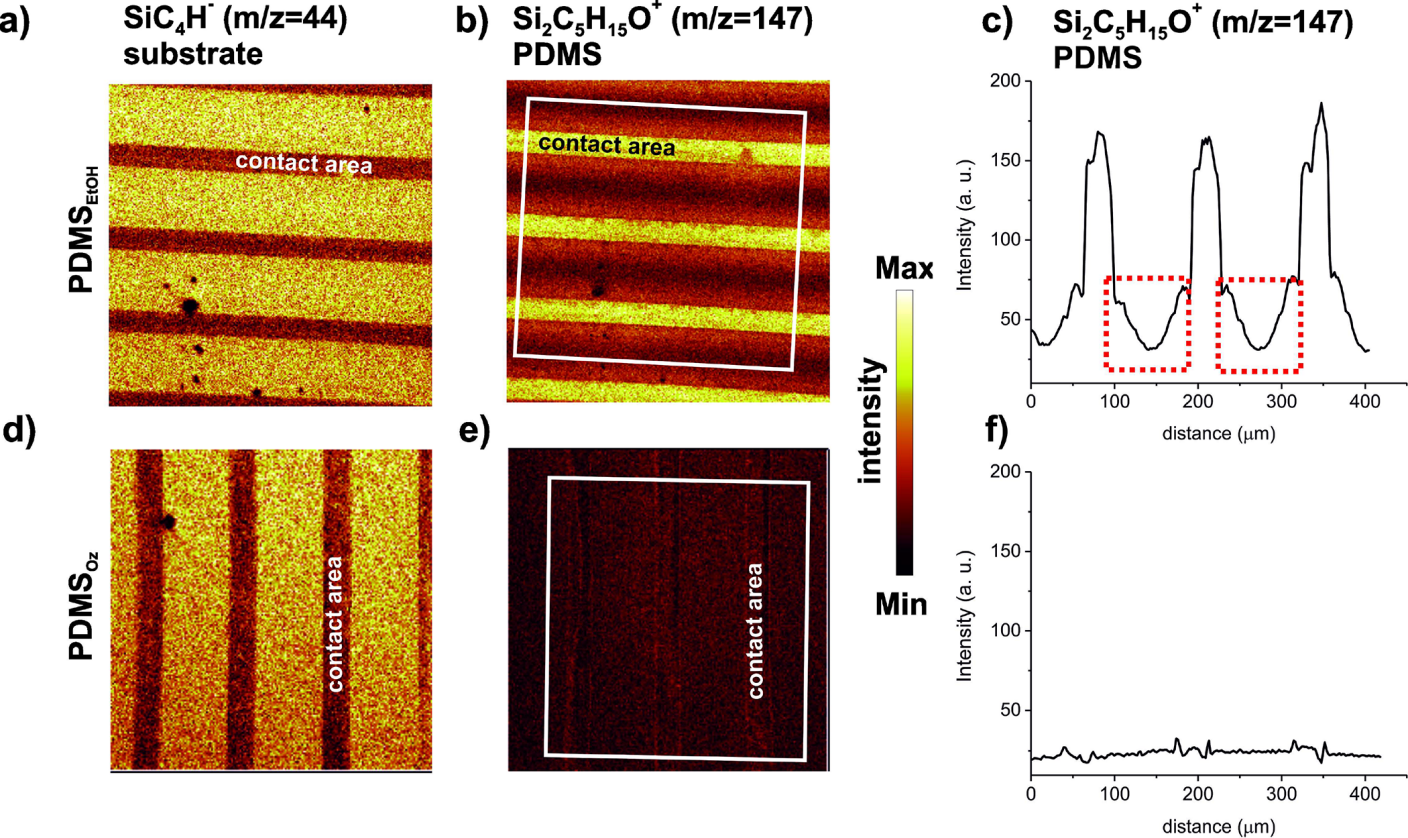
ToF-SIMS chemical imaging of the WGA patterns prepared by solvent (a–c) and ozone (c, d) cleaned PDMS stamp. Maps (500 μm × 500 μm) of ions characteristic of the APTES coated glass substrate (SiC_4_H^–^, *m*/*z* = 44, scale 0–25 counts), and PDMS (Si_2_C_5_H_15_O^+^, *m*/*z* = 147, scale 0–60 counts). The average of the intensity profiles of the Si_2_C_5_H_15_O^+^ (c, f) over areas of the sample marked by white rectangles (perpendicular to the pattern orientation) is shown in (b) and (e). Red rectangles mark signal intensity within no-contact areas.

We subsequently applied ToF-SIMS to characterize the chemical maps of patterned substrates of μCP lectins using microstructured PDMS_EtOH_ and PDMS_Oz_ stamps, first with an emphasis on quantifying possible PDMS transfer in the process. SIMS data reflected high intensities of the impurity peak Si_2_C_5_H_15_O^+^ at the areas of substrate-elastomer stamp contact (PDMS_EtOH_Figure 7a,b). In addition, the intensity gradient of the signal is visible at regions of the triple interfaces (inked PDMS/air/substrate), marked by red rectangles in Figure 7c, which is in line with that previously reported for amino silanes stamped on fluoropolymer.^47^

**Figure 8:**
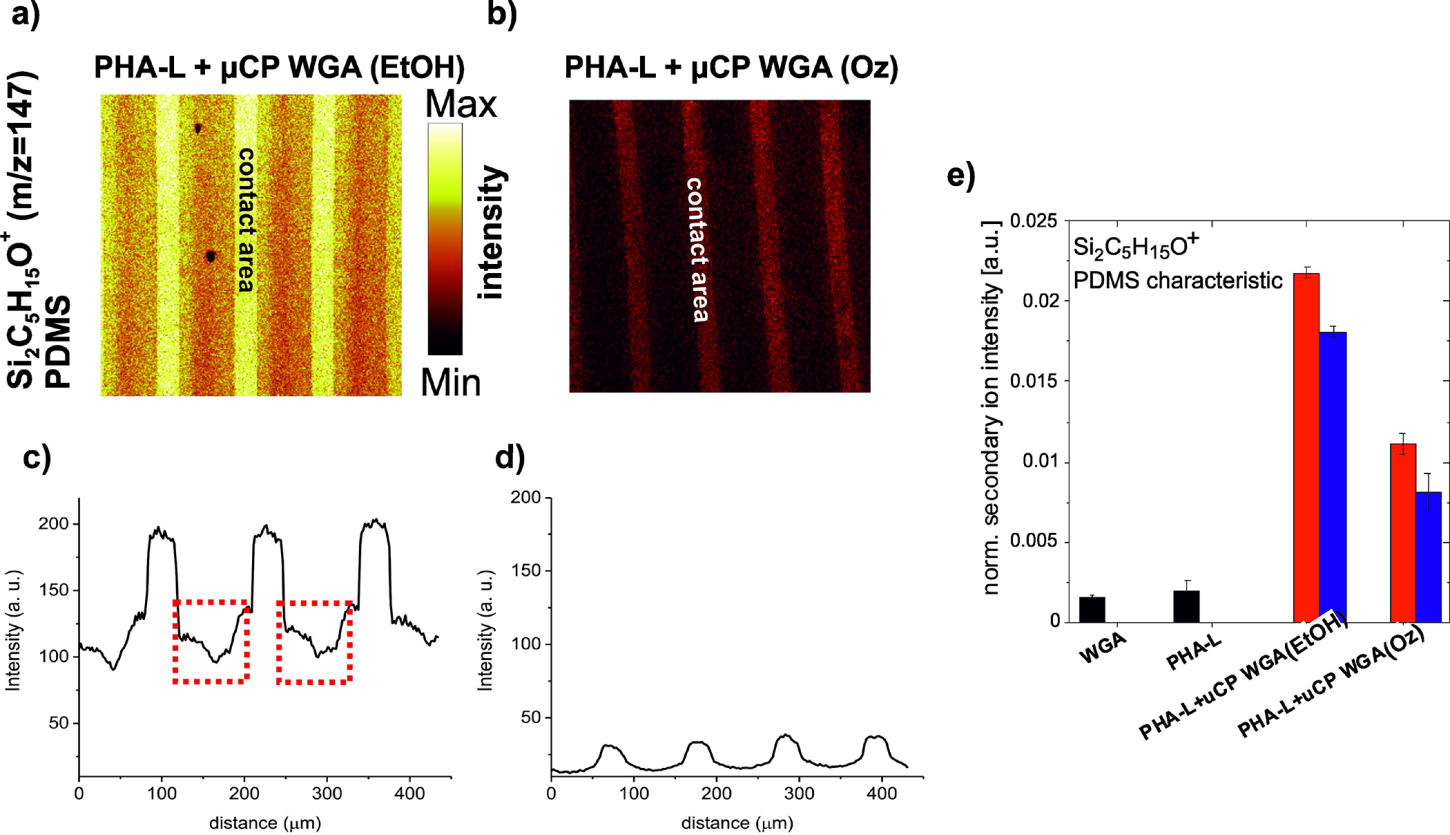
ToF-SIMS chemical imaging of transferred PDMS to dual-lectin patterns prepared by solvent (EtOH)- and ozone-cleaned elastomer stamps. Maps (500 μm × 500 μm) of ions characteristic for PDMS (Si_2_C_5_H_15_O^+^, *m*/*z* = 147, scale 0–60 counts) are shown. (c, d) Intensity profiles of Si_2_C_5_H_15_O^+^ over areas are shown in (a) and (b), respectively. Red rectangles mark ion concentrations of Si_2_C_5_H_15_O^+^ in the regions between the contact areas. (e) Intensities of positive ions characteristic for PDMS (Si_2_C_5_H_15_O^+^, *m*/*z* = 147) within stamp contact (red) and no contact (blue) areas are presented. Ion intensities from drop-cast layers of WGA and PHA-L proteins are added as references (black).

At variance with the PDMS transfer determined for μCP with PDMS_EtOH_, there is only a weak contrast in the image of the Si_2_C_5_H_15_O^+^ distribution when using PDMS_Oz_ in the μCP (Figure 7e,f). A marginally higher intensity of the signal can be seen at the contact regions (Figure 7f). Additionally, we investigated the effect of silicone contamination during the preparation of the dual-lectin pattern. Intensities of positive ions characteristic for PDMS (Si_2_C_5_H_15_O^+^, *m*/*z* = 147) within dual-lectin patterns produced by solvent- and ozone-cleaned stamps are shown in Figure 8a,b. Corresponding intensity profiles are plotted in Figure 8c,d. Interestingly, next to a substantial PDMS transfer observed within contact areas, there is also a substantial presence of PDMS observed in the regions between the contact regions when using PDMS_EtOH_ stamps (Figure 8a,c).

Additionally, in Figure 8e, normalized intensities of *m*/*z* = 147 signals within stamp contact (red) and no contact (blue) areas are presented. The intensities of the Si_2_C_5_H_15_O^+^ signal are about 50% lower when the PDMS_Oz_ stamp was used (Figure 8e), 2.18(4) × 10^–2^ (imprinted WGA (contact), PDMS_EtOH_) and 1.11(7) × 10^–2^ (imprinted WGA (contact), PDMS_Oz_)).

## 4 Discussion

Protein adsorption and microcontact printing are widely used in a variety of bioapplications. Both approaches have inherent challenges that affect the functionality of the protein-coated substrates. The lack of control over protein orientation is a shared common drawback in both protein adsorption and μCP.^22,53^ Additionally, this depends on experimental conditions such as lectin deposition time or substrate-stamp contact time.^54^ It is important therefore to know the structure of the lectin and to optimize exposition times. In this study, we used two lectins. Both lectins are composed of identical monomers. In this case, there is less chance that random molecular orientation of the lectin molecule on the substrate will result in a reduced number of functional binding sites. In addition, Hoang et al.^55^ demonstrated by binding capacity of dextran to Con A covalently attached to a quartz surface that the density of active immobilized Con A was about 1/3 of the theoretical maximum surface coverage estimate. Previous studies suggest that adsorption to hydrophobic surfaces does not drastically reduce lectin sugar binding capacity,^56^ and that 15 min lectin deposition time is sufficient to obtain homogeneously covered substrates.^52,56^ Both approaches result in the formation of lectin layers of different organization. Fluorescence images reveal higher fluorescence intensity, indicating higher number of lectins, in the case of DC. In addition, a close inspection of fluorescence images of lectin layers imprinted with PDMS stamps shows a terraced structure of imprinted layers (see Figures S2 and S3). This indicates that during DC, lectins form multilayers. This is in agreement with previous report by Mielczarski et al.^57^

In the case of μCP, an additional drawback is the potential leakage of elastomeric contaminants that occurs during the printing of the lectin. Although several research groups reported silicone oligomers transfer previously,^1,30,46−50^ only Foley et al.^1^ investigated its influence on the binding capacity of imprinted IgG and its antibodies. We are the first to study cell adhesion to lectin layers imprinted with PDMS stamps treated with different solvents and UV ozone plasma. We have observed a significant decrease in the number of cells adhering to the lectin layers that were imprinted with PDMS_EtOH_ stamps. The reduced number of adhered cells and adhesion strength were confirmed by static adhesion assays and single-cell force spectroscopy measurements, respectively. Our data suggest that one of the main factors responsible for the low and weak adhesion of cells is elastomeric contaminants transferred during the microcontact printing of lectins. ToF-SIMS data revealed a significant amount of PDMS on the surface of protein layers prepared by μCP. The amount of transferred silicone oligomers changes chemical properties of the substrate, which becomes more hydrophobic,^46^ whereas cells adhere preferably to hydrophilic substrates. And so the reduction in cell adhesion to PDMS contaminated substrates should be expected. Knowing that PDMS-contaminants harm the binding capacity of the imprinted lectins (compared to physical adsorption of proteins, DC), we tested a different PDMS cleaning protocol based on the short-time exposure of stamps to UV ozone plasma before protein deposition. Static adhesion experiments and chemical characterization (ToF-SIMS) of the microcontact printed lectin layers indicate that applying plasma activation instead of solvent cleaning reduces silicone transfer significantly.

Alongside the evidence presented here on explicitly determining that the UV ozone pretreatment of the PDMS stamps reduces the transfer of silicone on μCP, the possibility that there are differences in lectin denaturation in contact with differently processed PDMS cannot be entirely ruled out. Based on the possibility that PDMS hydrophobic surfaces induce denaturation^58,59^ and that UV ozone pretreatment of PDMS reduces hydrophobicity with associated effect of reducing extent of denaturation, this facet may also be a possible contributing mechanism for increased cell adhesion to lectin surfaces prepared by μCP (PDMS_Oz_) as compared to PDMS_EtOH_.

Our results show that control of surface chemistry is needed when μCP is applied in the fields of biosensors and bionanotechnology. Still, no reliable protocol for microcontact printing of biomolecules that guarantees low silicone contamination is established. Our research and literature data show that PDMS polymerization conditions and stamp treatment (age, cleaning, and storage conditions) prior to the deposition and transfer of proteins are pivotal for the binding capacity of imprinted biomolecules. Swelling and sol fraction experiments showed that PDMS contain free oligomers, which can be transferred during μCP.

When comparing the curing conditions of PDMS stamps used by different research groups, different curing temperatures or/and durations are used.^42,60−62^ Also, various alcohols or their water dilutions are used to remove contaminants from the surface of the elastomer stamps. It is a minority who apply plasma cleaning to remove contaminant material (proteins, dirt, etc.). Although data suggest that UV ozone plasma treatment of PDMS stamps reduces silicone transfer, still further research is needed to identify the mechanisms and quantify the transfer of silicone oligomers to the substrate from stamps activated with different types of plasma (O_2_, N_2_, H_2_, air). Such studies are needed to optimize the preparation and handling of elastomer stamps used for microcontact printing of biomolecules.

We also address the issue of silicone contamination occurring during single- and dual-lectin pattern preparation. ToF-SIMS data revealed that in the case of microstructured elastomer stamps cleaned with EtOH, high intensity of PDMS characteristic ions (Si_2_C_5_H_15_O^+^) was observed within areas in contact with PDMS stamp. The intensity gradient of the Si_2_C_5_H_15_O^+^ ions was also noted close to the PDMS–substrate contact region. Contrary to that, in the case of UV ozone-treated PDMS stamps, a much lower intensity of Si_2_C_5_H_15_O^+^ ions and no silicone cross-contamination of the surface below stamp grooves were registered. The results indicate that short-term UV ozone treatment of PDMS stamps inhibits silicone contamination of imprinted lectin layers and so does not interfere with lectin binding-capacity.

## 5 Conclusions

Here we report that the preprocessing of PDMS stamps used for the μCP of biomolecules is critical to their functionality. We show that solvent-based cleaning of PDMS stamps is detrimental to the quality and functionality of biopatterns. Silicone residues transferred during μCP (solvent cleaning approach) inhibited the adhesion of bladder cancer cells to imprinted layers of high-affinity lectins. Reduced PDMS transfer and restoration of biopattern functionality were achieved by a short-time UV ozone treatment of the PDMS stamp. We also show that the combination of drop casting and μCP techniques allows the fabrication of dual-lectin patterns that can be used in many biotechnological applications.

## Supporting Information Available

Weight change data of PDMS cuboids before and after swelling and drying; images showing changes of wettability of PDMS after plasma exposure; descriptions of protocols used for μCP of lectins with PDMS stamps pretreated with cyclohexane; representative fluorescence images of lectin layers deposited by drop-casting and transferred with PDMS stamps; results of the quantitative analysis of fluorescence intensity of lectin layers; bladder cancer cells adhesion to lectin layers imprinted with solvent cleaned (CHX and EtOH) PDMS stamps; representative force–distance curves obtained during SCFS experiments and distributions of adhesion work values for individual probe cells; schemes showing single- and dual-lectin micropatterns production; section on the quality of lectin-based micropatterns assessed by ToF-SIMS (PDF)
