# Supplementary material for: Plasma Treatment of PDMS for Microcontact Printing (μCP) of Lectins Decreases Silicone Transfer and Increases the Adhesion of Bladder Cancer Cells
Source: ACS Appl Mater Interfaces. 2023 Oct 27;15(44):51863–75. doi: 10.1021/acsami.3c09195 (PMC10636731; doi:10.1021/acsami.3c09195)
Supplement: Supplementary file 1 — am3c09195_si_001.pdf [file am3c09195_si_001.pdf]

Supporting Information

for

Plasma treatment of PDMS for microcontact printing (μCP) of lectins decreases silicone transfer and increases the adhesion of bladder cancer cells

Joanna Zemła[1,], Renata Szydlak[1], Katarzyna Gajos[2], Łukasz Kozłowski[1], Tomasz Zieliński[1], Marcin Luty[1], Ingrid H. Øvreeide[3], Victorien E. Prot[4], Bjørn T. Stokke[3,*], Małgorzata Lekka[1,*]*

[1] Institute of Nuclear Physics, Polish Academy of Sciences, PL-31342 Krakow, Poland

[2] M. Smoluchowski Institute of Physics, Jagiellonian University, 30348 Kraków, Poland

[3] Biophysics and Medical Technology, Department of Physics, The Norwegian University of Science and Technology (NTNU), NO-7491 Trondheim, Norway

[4] Biomechanics, Department of Structural Engineering, The Norwegian University of Science and Technology (NTNU), NO-7491 Trondheim, Norway

*corresponding authors:

joanna.zemla@ifj.edu.pl

malgorzata.lekka@ifj.edu.pl

bjorn.stokke@ntnu.no

Table S1 Determination of the sol fraction. Changes in mass of three PDMS pieces after swelling in EtOH and drying. Data presented as mean ± maximum error (0.0001 g + SD).

m [g]	**PDMS 01**	**PDMS 02**	**PDMS 03**
PDMS	0.2434 ± 0.0003	0.2732 ± 0.0002	0.2274 ± 0.0002
Swollen PDMS	0.2491 ± 0.0004	0.2801 ± 0.0006	0.2340 ± 0.0005
Dried PDMS	0.2360 ± 0.0004	0.2647 ± 0.0004	0.2205 ± 0.0004
Sol fraction	**0.0074 ± 0.0006**	**0.0086 ± 0.0005**	**0.0069 ± 0.0006**
	**3.0 %**	**3.1 %**	**3.0 %**

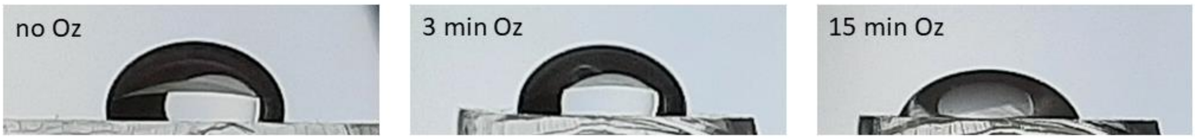

Figure S1 100 µL drop of ultra-pure water deposited on PDMS cuboids before and after UV ozone treatment.

µCP of lectins with flat PDMS stamps sonicated in cyclohexane

A set of substrates covered with PHA-L and WGA imprinted with cyclohexane (CHX) treated PDMS cuboids were prepared. The flat PDMS stamps were sonicated in CHX for (15 min) and dH_2O (15 min), and dried with N_2. Subsequently, these stamps were used to deposit lectins on APTES-functionalized glass slides following the protocol described in Section 2.8.

Analysis of fluorescence micrographs reflecting lectin distribution

Fluorescence microscopy (see Section 2.11 for details) was used to visualize and compare lectin layers prepared with different protocols (DC, µCP with $PDMS_{Oz}$, µCP with $PDMS_{EtOH}$, and µCP with $PDMS_{CHX}$). The camera acquisition time was 6 ms for all lectin types. For each case, 3 substrates were prepared and a minimum of 6 images were taken. The

distribution of fluorescence intensity and its mean ± SD of each image were obtained using CellSens software (Olympus). Mean fluorescence intensities were then calculated for all data sets and normalized to the background signal (glass + APTES). Figures S2 and S3 show representative fluorescence images of WGA-TRITC and PHA-L-FITC layers, and normalized fluorescence intensities obtained for drop-cast and imprinted lectin layers.

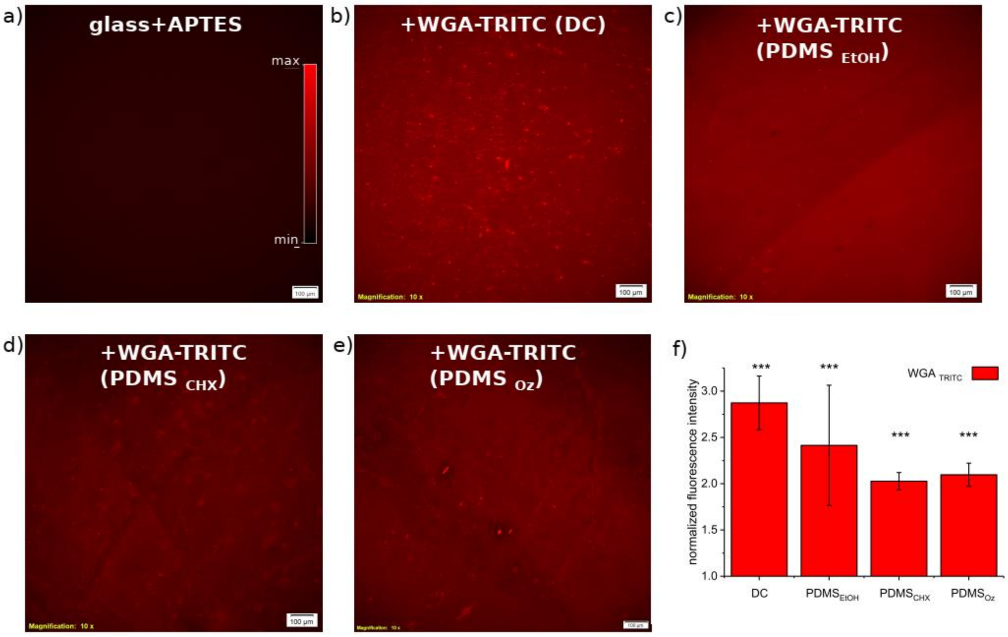

Figure S2 Representative fluorescence images of the substrate (a) and WGA-TRITC lectins deposited by DC (b) and μCP with flat PDMS stamps pretreated with EtOH (c), cyclohexane (CHX) (d) and UV ozone (e). In (a), bar showing fluorescence intensity scale corresponding to images a - e. In (f), fluorescence intensities of deposited lectins normalized to the background signal (glass + APTES). Data presented as means ± SD. Statistical significance was determined with the t-test stating statistical significance between fluorescence intensity of lectin layers and the reference (glass + APTES). Scale bar 100 μm.

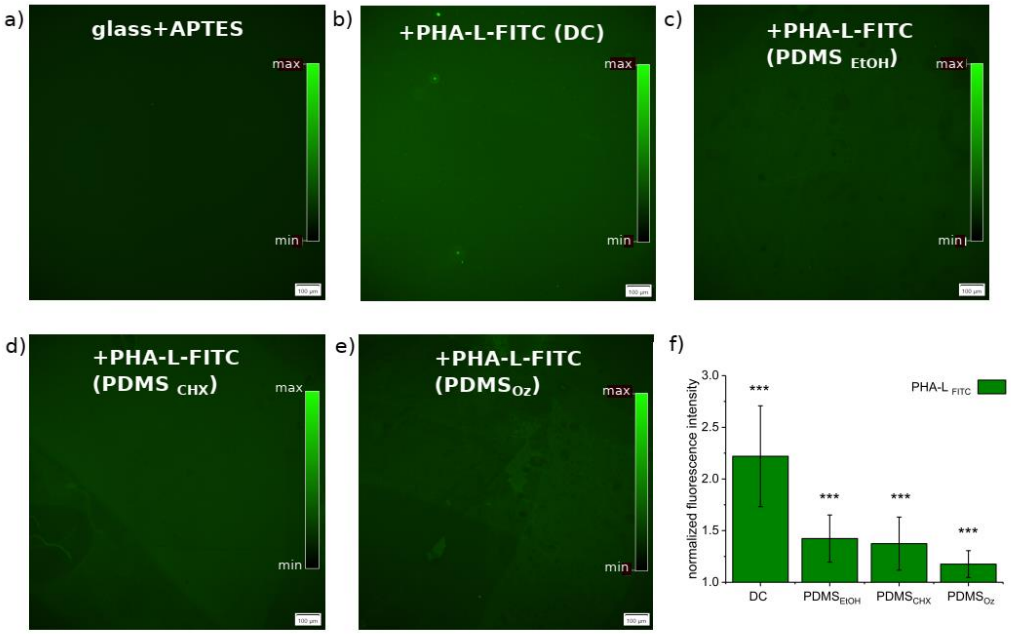

Figure S3 Representative fluorescence images of the substrate (a) and PHA-L-FITC lectins deposited by DC (b) and µCP with flat PDMS stamps pretreated with EtOH (c), cyclohexane (CHX) (d) and UV ozone (e). In (a), bar showing fluorescence intensity scale corresponding to images a - e. In (f), fluorescence intensities of deposited lectins normalized to the background signal (glass + APTES). Data presented as means ± SD. Statistical significance was determined with the t-test stating statistical significance between fluorescence intensity of lectin layers and the reference (glass + APTES). Scale bar 100 µm.

Bladder cancer cells adhesion to lectin layers imprinted with solvent-cleaned (CXH and EtOH) PDMS stamps.

Figure 4S shows mean number of HCV29 and HT1376 cells per 1 mm^2 on lectin layers deposited by drop-casting or transferred on the APTES-coated glass slides with $PDMS_{EtOH}$ and $PDMS_{CHX}$ stamps. As it was expected, a decreased number of cells adhered onto substrates prepared with solvent-cleaned elastomers is observed. There is no statistically significant difference between the number of HCV29 cells on imprinted lectin layers (30 ± 11 cells/mm^2 (CHX) vs 39 ± 12 cells/mm^2 (EtOH)). Similar is observed for HT1376 cells (9.2 ± 2.6 cells/mm^2 (CHX) vs 10.1 ± 1.4 cells/mm^2 (EtOH)). Most abundant cell adhesion is observed in the case

of drop-cast substrates, 235 ± 33 cells/mm^2 and 48.4 ± 7.7 cells/mm^2, HCV29 and HT1376 cells, respectively.

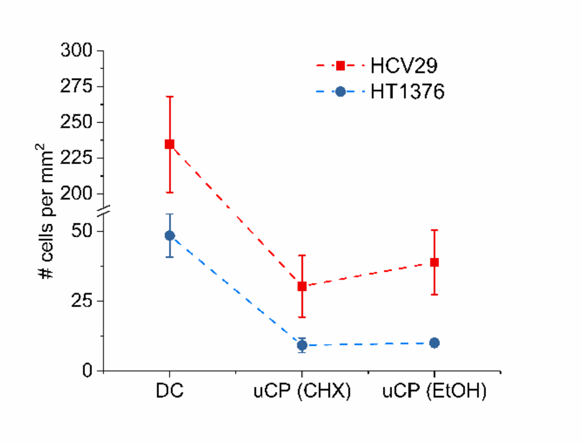

Figure S4 Adhesion of HCV29 and HT1376 cells to PHA-L and WGA-modified substrates prepared using DC or μCP. Data presented as mean ± SD.

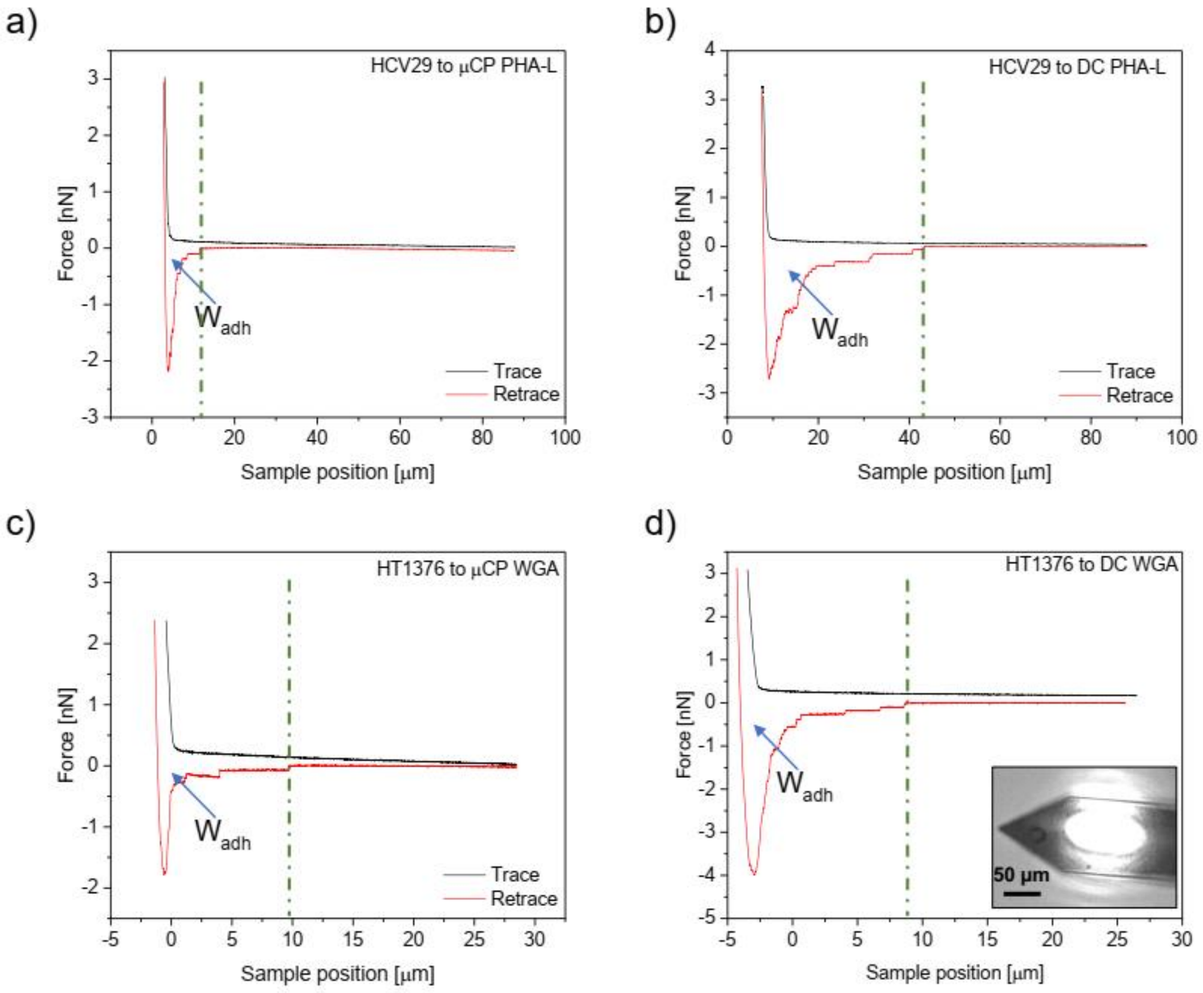

Figure S5 Representative force-distance curves obtained during SCFS. Interaction of HCV29 probing cell with PHA-L layer deposited by micro-contact printing with $PDMS_{EtOH}$ (a), and physical adsorption (b). Interaction of HT1376 with WGA molecules deposited by µCP ($PDMS_{EtOH}$) (c), and DC (d). In the inset of (d), the optical view of the probing cell attached to the Con A-functionalized probe. Vertical lines depict the last unbinding event, which is used as the upper boundary of the cell-lectin interaction in the calculation of the adhesion.

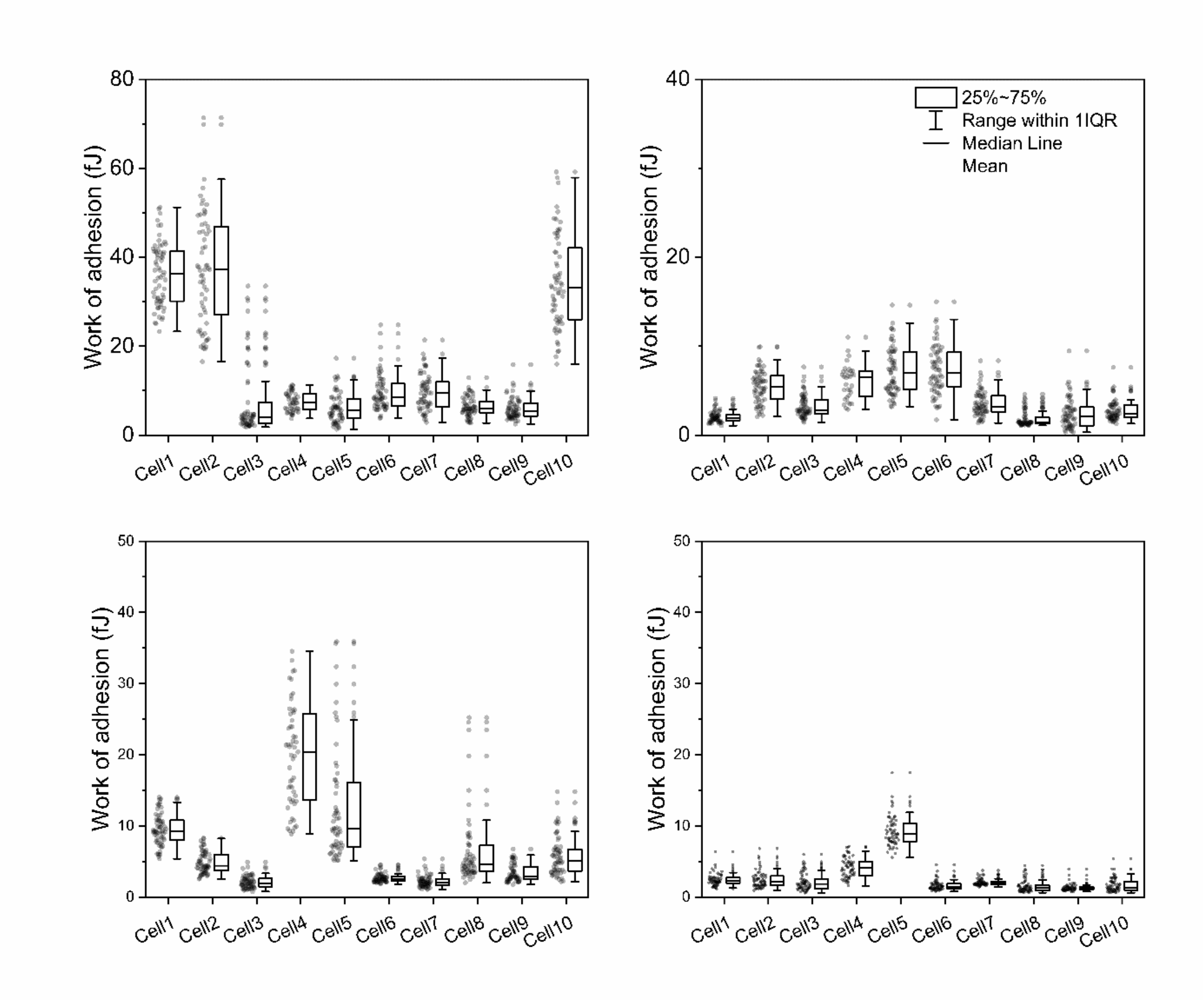

Figure S6 Distribution of the values of the adhesion work for HCV29 (top row) and HT1376 (bottom row) cells detached from PHA-L and WGA-coated surfaces. Lectins were deposited with DC (left column) and µCP (right column). Flat $PDMS_{EtOH}$ stamps were used.

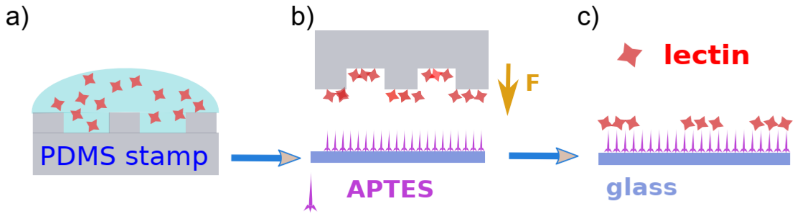

Figure S7 Schematic illustration of printing of lectin patterns with a PDMS stamp.

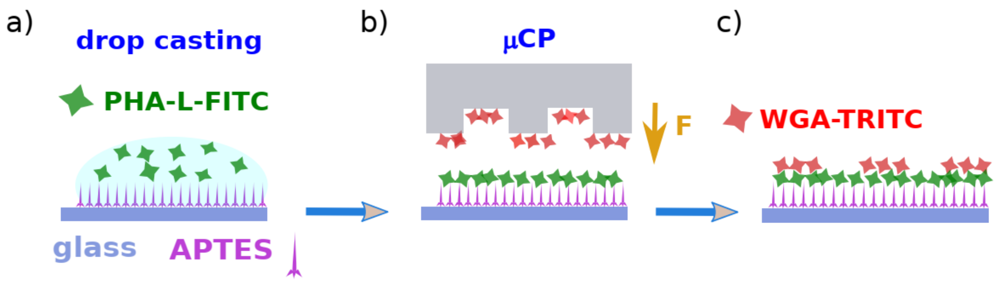

Figure S8 Schematic illustration of dual-lectin patterns preparation. Elastomer stamp is used to transfer WGA-TRITC lectins onto drop-cast PHA-L-FITC layer on an APTES functionalized glass surface.

Quality of lectin-based micropatterns assessed by ToF-SIMS

To verify the chemical selectivity of the single and dual-lectin patterns, a number of characteristic m/z signals specific for the lectins were identified. ToF-SIMS was used to investigate the amino acid composition of the WGA and PHA-L lectins from measurements for one-component drop-cast layers. The normalized intensities of selected peaks from each spectrum are shown in Table S2. In the case of WGA, high intensities of ion peaks corresponding to the amino acids: Alanine (Ala), Histidine (His), Glutamic Acid (Glu), and Glutamine (Gln) were found. PHA-L showed a higher concentration of Arginine (Arg), Serine (Ser), Valine (Val), Threonine (Thr), Isoleucine (Ile), Leucine (Leu), Phenylalanine (Phe), and Tryptophan (Trp). The highest intensity contrast was given by C_4H_6NO+ and $C_5H_{12}N+$ ion peaks characteristic of Glu/Gln and Ile/Leu, respectively. In the case of WGA, the intensity of the C_4H_6NO+ ion was higher than in the PHA-L spectrum. The signal ratio ($C_4H_6NO+_{WGA}$/ $C_4H_6NO+_{PHA\text{-}L}$) was of 1.6. The intensity of the characteristic signal of the Ile/Leu was ~3-folds higher for PHA-L than WGA . Both lectins showed a high and comparable (4.75(10) 10^{-2} and 4.01(6) 10^{-2}) intensity of the Lysine (Lys) signal ($C_5H_{10}N+$, m/z = 84).

Table S2 ToF-SIMS examination of the amino acids composition of the WGA and PHA-L lectins. Comparison of the intensity of secondary ions derived from different amino acids acquired for WGA and PHA-L layers drop cast on APTES-modified glass substrates. The intensity of selected peaks from each spectrum was normalized to the sum of amino acids derived ions.

Amino acid	Ion peaks	WGA intensity [a.u]	PHA-L intensity [a.u]
**Gly**	CH_4N^+ m/z=30	1.57(4)E-01	1.22(7)E-01
**Arg**	$CH_3N_2^+$ m/z=43	3.74(15)E-02	4.73(6)E-02
**Ala**	$C_2H_6N^+$ m/z=44	2.48(13)E-01	1.50(26)E-01
**Ser**	$C_2H_6NO^+$ m/z=60	4.80(6)E-02	6.70(10)E-02
**Pro**	$C_4H_6N^+$ m/z=68	3.20(5)E-02	2.82(10)E-02
**Asn**	$C_3H_4NO^+$ m/z=70	1.97(4)E-02	2.20(15)E-02
**Pro**	$C_4H_8N^+$ m/z=70	1.49(4)E-01	1.49(8)E-01
**Ser**	$C_3H_3O_2^+$ m/z=71	3.70(8)E-03	7.74(6)E-03
**Val**	$C_4H_{10}N^+$ m/z=72	2.03(2)E-02	6.84(12)E-02
**Thr**	$C_3H_8NO^+$ m/z=74	7.84(13)E-03	2.58(4)E-02
**His**	$C_4H_5N_2^+$ m/z=81	2.13(3)E-02	1.20(6)E-02
**His**	$C_4H_6N_2^+$ m/z=82	2.09(3)E-02	1.32(5)E-02
**Val**	$C_5H_7O^+$ m/z=83	9.42(16)E-03	1.95(7)E-02
**Glu, Gln**	$C_4H_6NO^+$ m/z=84	3.63(8)E-02	2.28(4)E-02
**Lys**	$C_5H_{10}N^+$ m/z=84	4.75(10)E-02	4.01(6)E-02
**Ile, Leu**	$C_5H_{12}N^+$ m/z=86	2.45(2)E-02	7.30(10)E-02
**Asn**	$C_3H_7N_2O^+$ m/z=87	1.32(2)E-02	1.41(3)E-02
	$C_3H_6NO_2^+$ m/z=88	6.85(5)E-03	7.09(6)E-03
	$C_4H_4NO_2^+$ m/z=98	4.61(5)E-03	5.30(20)E-03
**Arg**	$C_4H_{10}N_3^+$ m/z=100	9.30(8)E-03	1.16(2)E-02
**Glu**	$C_4H_8NO_2^+$ m/z=102	8.21(11)E-03	7.02(19)E-03
**His**	$C_5H_8N_3^+$ m/z=110	3.26(4)E-02	1.39(2)E-02
**Phe**	$C_8H_{10}N^+$ m/z=120	1.77(2)E-02	4.34(3)E-02
**Arg**	$C_5H_{11}N_4^+$ m/z=127	3.94(4)E-03	4.51(9)E-03
**Trp**	$C_9H_8N^+$ m/z=130	1.57(3)E-02	1.69(4)E-02
	$C_{10}H_{11}N_2^+$ m/z=159	2.87(6)E-03	4.16(14)E-03
	$C_{11}H_8NO^+$ m/z=170	3.17(6)E-03	4.25(6)E-03

The surface distribution of WGA lectin within a single lectin pattern is shown in Figure S9. ToF-SIMS data confirmed selective protein deposition independent of stamp preparation protocols.

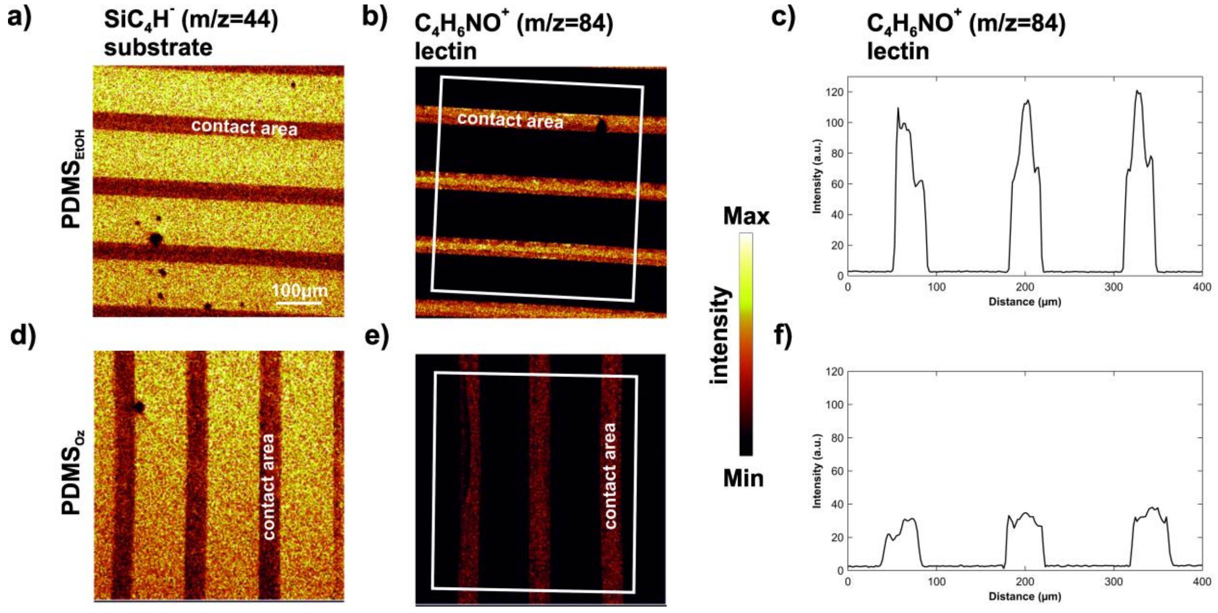

Figure S9 ToF-SIMS analysis of lectin distribution in single-lectin micropatterns prepared by $PDMS_{EtOH}$ or $PDMS_{Oz}$ stamps (a-c) and (d-f), respectively. Maps (500 µm × 500 µm) of characteristic ions corresponding to the substrate (SiC_4H^-, *m/z* = 44, scale 0 - 25 counts) (a, d) and WGA ($C_4H_6NO^+$ of Glu and Gln, *m/z* = 84, scale 0 - 30 counts) (b, e) are shown. In (c) and (f), an average of intensity profiles (perpendicular to the pattern orientation) over the areas marked with white squares in (b) and (e) is shown, respectively.

Comparison of $C_4H_6NO^+$ ion intensities indicate a uniform distribution of biomolecules within the pattern (Fig. S9c, f). Maps of the $C_4H_6NO^+$ signal from Glu and Gln amino acids (Table S2) showed similar patterns to fluorescence images of the WGA distribution. However, the Glu/Gln signal is lower for the WGA pattern imprinted with the ozone-cleaned stamp. This observation is not consistent with the fluorescence images and may be due to the matrix effect in the ToF-SIMS analysis, which may be caused by the different composition of silicone oligomer contamination. UV ozone plasma treatment of PDMS (exposure time ≤ 30 min) results in surface oxidization and high segmental mobility of PDMS, reflected in relatively hydrophobic surfaces exhibiting a high water contact-angle hysteresis[1].

In the next step, we deposited two types of lectins (one by DC and the other by µCP) to form dual-lectin micropatterns. Analogous to the single-lectin micropatterns, the ToF-SIMS analysis was performed to identify the deposited lectins and verify the selectivity of the lectin patterning (Figure S10a).

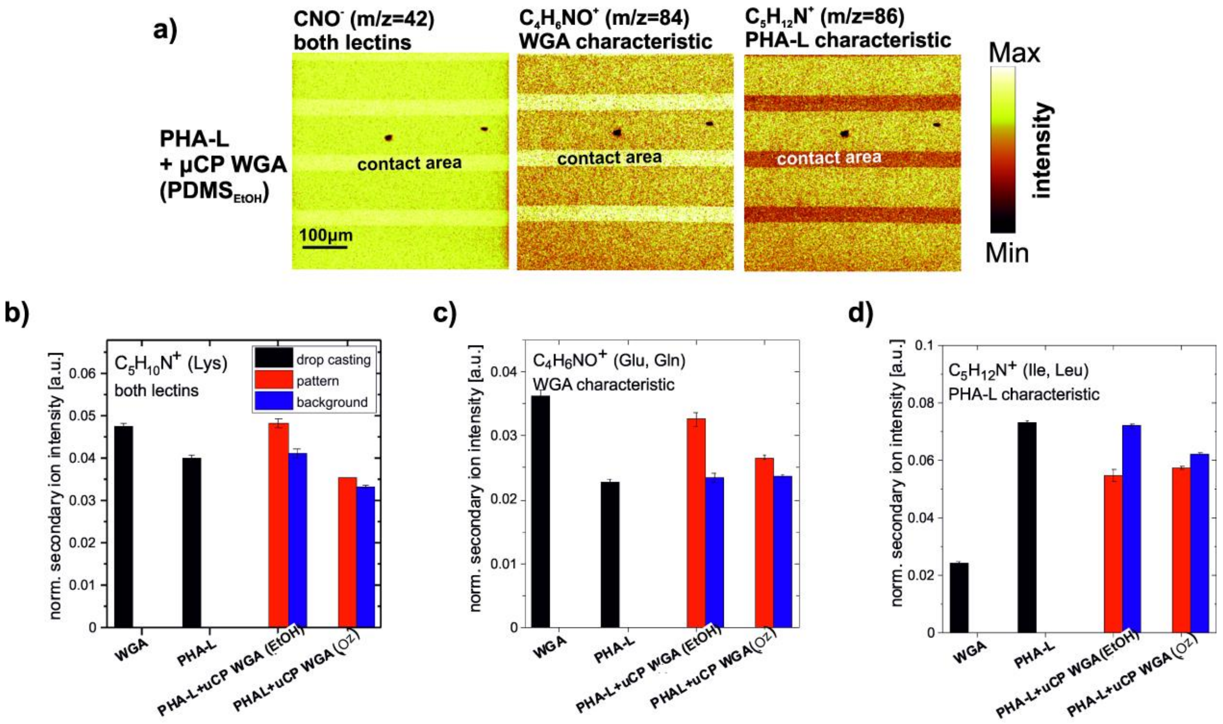

Figure S10 ToF-SIMS analysis of dual-lectin patterns. (a) ToF-SIMS chemical imaging of WGA and PHA-L distribution. Maps (500 µm × 500 µm) of characteristic ions corresponding to both lectins (CNO−, m/z = 42, scale 0 - 130 counts), WGA ($C_4H_6NO^+$ of Glu and Gln, m/z = 84, scale 0 - 30 counts) and PHA-L ($C_5H_{12}N^+$ of Ile and Leu, m/z = 86, scale 0 - 35 counts) are presented. (b-d) Analysis of molecular surface composition based on high mass resolution ToF-SIMS spectra for dual lectin patterns prepared by EtOH ($PDMS_{SC}$) and UV ozone ($PDMS_{Oz}$) -cleaned PDMS stamps. ToF-SIMS spectra were acquired selectively in the area of the WGA stripe (pattern, red) and PHA-L layer (background, purple). Intensities of positive ions characteristic for both lectins ((b), $C_5H_{10}N^+$ of Lys, *m/z* = 147), WGA ((c), $C_4H_6NO^+$ of

Glu and Gln, *m/z* = 84) and PHA-L ((d), $C_5H_{12}N+$ of Ile and Leu, *m/z* = 86). Ion intensities from drop-cast layers of WGA and PHA-L proteins (black) are added as references.

ToF-SIMS provided a detailed analysis of the WGA and PHA-L distribution, namely the intensity distribution of C_4H_6NO+ and $C_5H_{12}N+$ ions. It confirmed the surface composition illustrated by fluorescence images of WGA/PHA-L patterns, which showed the regular pattern of alternating broad and narrow stripes of protein domains. It also confirmed the homogeneous distribution of biomolecules within the lectin domains. The highest intensity of the C_4H_6NO+ signal was registered for WGA domains, corresponding to areas in contact with a PDMS stamp, whereas the PHA-L characteristic signal intensity is prominent within regions under the stamp's grooves. It can be concluded that the imprinted WGA lectins cover a lower PHA-L layer to a large extent. This is also confirmed by a detailed analysis of the normalized signal intensities shown in Fig. S10b-d. An increased intensity of the Glu/Gln signal is observed in the WGA regions (Fig. S10c, red), and at the same time Ile/Leu characteristic peaks are of greater intensity when examining the 'no-contact' stamp area (Fig. S10d, purple).

References

(1) Hillborg, H.; Tomczak, N.; Olàh, A.; Schönherr, H.; Vancso, G. J. Nanoscale Hydrophobic Recovery: A Chemical Force Microscopy Study of UV/Ozone-Treated Cross-Linked Poly(Dimethylsiloxane). *Langmuir* **2004**, *20* (3), 785–794. https://doi.org/10.1021/la035552k.

(2) Zemła, J.; Lekka, M.; Wiltowska-Zuber, J.; Budkowski, A.; Rysz, J.; Raczkowska, J. Integral Geometry Analysis of Fluorescence Micrographs for Quantitative Relative Comparison of Protein Adsorption onto Polymer Surfaces. *Langmuir* **2008**, *24* (18). https://doi.org/10.1021/la801313u.
